# Efficacy and safety of upadacitinib for patients with immune-mediated inflammatory diseases: a systematic review and meta-analysis

**DOI:** 10.3389/fimmu.2025.1586792

**Published:** 2025-07-01

**Authors:** Rui Chai, Xiaomin Li, Wei Shen, Ziyi Jin, Genhong Yao, Xiaojun Tang, Linyu Geng, Lingyun Sun

**Affiliations:** ^1^ Department of Rheumatology and Immunology, Nanjing Drum Tower Hospital Clinical College of Nanjing Medical University, Nanjing, Jiangsu, China; ^2^ Department of Rheumatology and Immunology, The Affiliated Drum Tower Hospital of Nanjing University Medical School, Nanjing, Jiangsu, China

**Keywords:** immune-mediated inflammatory disease, upadacitinib, JAK inhibitors, systematic review, meta-analysis

## Abstract

**Objective:**

There is a growing array of options for the treatment of immune-mediated inflammatory diseases (IMIDs). To explore upadacitinib’s efficacy and safety in autoimmune disease treatment, we conducted this study.

**Methods:**

Pubmed, Web of Science and Embase were searched for randomized controlled trials related to the treatment of upadacitinib from the databases’ inception to May 31, 2024. After literature screening, data extraction and bias assessment by two investigators, RevMan 5.3 or Stata 17.0 software was used for meta-analysis.

**Results:**

45 records across the following five types of IMIDs were obtained. For rheumatoid arthritis (RA), upadacitinib 15 mg outperformed placebo, methotrexate and adalimumab (ADA) in 20% improvement according to ACR criteria (ACR20) and 28-joint disease activity score (DAS28) (*P* < 0.05). It also improved quality of daily life based on pain relief, morning stiffness and 36-Item Short Form Health Survey, etc. For axial spondyloarthritis (axSpA), upadacitinib 15 mg enhanced 20/40% improvement in Assessment of SpondyloArthritis international Society (Risk Ratio [RR] = 1.28/1.47), with better rates of low disease activity and inactive disease as well. For psoriatic arthritis (PsA), upadacitinib 15 mg or 30 mg significantly improved ACR20 compared to placebo (RR = 2.46/2.68, *P* < 0.001) and reduced psoriasis skin lesions, though it showed no superior benefit for enthesitis compared to placebo. For Crohn’s disease (CD), upadacitinib 45 mg significantly improved stool frequency and abdominal pain score clinical remission compared to placebo (RR = 2.47, 95% CI [2.12, 2.88], *P* < 0.001) as well as Crohn’s Disease Activity Index score remission and endoscopic response (*P* < 0.001). For ulcerative colitis (UC), upadacitinib 45 mg increased clinical remission rates (RR = 6.92, 95% CI [4.99, 9.59], *P* < 0.001) and improved symptoms like bowel frequency and abdominal pain (*P* < 0.05). Overall adverse events (AEs) rates were generally similar to non-upadacitinib groups (RR = 1.02, 95% CI [0.98, 1.07]). However, the higher risks of infections especially herpes zoster (HZ) must be highlighted in upadacitinib group. Although the incidence of death, serious adverse events (SAEs), and long-term risks like cardiovascular events and malignancies were without statistic significant differences, careful monitoring during treatment would still be essential.

**Conclusions:**

Upadacitinib is effective in treating IMIDs like RA, axSpA, PsA, CD, and UC. Though well-tolerated generally, its safety in infection especially HZ needs caution. Thorough assessment, monitoring and individualized dosing are vital to manage potential AEs.

**Systematic Review Registration:**

https://www.crd.york.ac.uk/prospero/, identifier CRD42024569370.

## Introduction

Immune-mediated inflammatory diseases (IMIDs) represent a diverse group of diseases characterized by chronic inflammation and organ damage, with varying manifestations depending on the organs primarily affected. These diseases encompass gut-related inflammatory bowel diseases (IBD) such as Crohn’s disease (CD) and ulcerative colitis (UC), as well as joint-related like rheumatoid arthritis (RA), psoriatic arthritis (PsA), and axial spondyloarthritis (axSpA), etc. (1) Epidemiological studies indicate that the prevalence of immune-mediated diseases is increasing (2, 3).

Over the past two decades, the treatment for IMIDs has undergone a significant transformation. We have shifted from the use of broad-spectrum immune modulators to the widespread adoption of highly targeted therapies thanks to advancements in monoclonal antibody technologies, molecular biotechnology, and more recently, the application of highly targeted medicines like Janus kinase (JAK) inhibitors (4).

JAK inhibitors, as a type of small molecule targeted drug preparation with rapid onset and multi-target advantages, have been gradually accepted and applied in clinical practice, demonstrating good therapeutic effects (5–8). Upadacitinib is a novel selective JAK1 inhibitor that has been approved for the treatment of atopic dermatitis (AD), PsA, RA, AS, IBD including CD and UC, etc. (9).

Recently, many trials have been conducted to determine the efficacy and safety of upadacitinib, and it has been increasingly used by clinicians to treat immune-related diseases. However, most studies have focused on the efficacy and safety of upadacitinib in treating some specific IMIDs. In contrast, our study offers a timely synthesis of recent randomized controlled trials (RCTs), aiming to comprehensively evaluate the evidence and provide solid insights that can guide clinical decision-making and improve treatment outcomes for IMIDs.

## Methods

### Protocol

This systematic review and meta-analysis was conducted in accordance with protocols registered in the PROSPERO (CRD42024569370) and the PRISMA 2020 checklist (10) (**Supplementary Table S1**).

### Literature search

Three commonly used databases (Pubmed, Web of Science, and Embase) were searched for literature on upadacitinib in the treatment of IMIDs. The search period covered from the inception of the databases up to May 31, 2024. The search strategies are detailed in the table (**Supplementary Table S2**).

### Search criteria

#### Participants

Patients were diagnosed with immune-mediated diseases based on pre-established criteria. IMIDs included but were not limited to RA, axSpA including ankylosing spondylitis (AS) and non-radiographic axSpA (nr-axSpA), PsA, CD, and UC.

#### Intervention methods

The experimental group received upadacitinib therapy, either as monotherapy or in combination with other treatments, while the control group received therapy without upadacitinib. There were no restrictions on the dose of upadacitinib or the duration of the intervention.

#### Outcomes

We assessed efficacy outcomes using recognized criteria for IMIDs, such as clinical remission and endoscopic response for IBD, American College of Rheumatology (ACR) response criteria for RA and PsA, axial spondyloarthritis disease activity score (ASDAS) and bath ankylosing spondylitis disease activity index (BASDAI) for axSpA, and others. Adverse events (AEs) were also recorded and analyzed as key outcome measures.

#### Study design

All included trials were RCTs, either conducted as individual studies or as part of pooled analyses. There were no restrictions on the RCTs included.

### Exclusion criteria

1. The type of target literature did not match, including reviews, case reports, guidelines, conference abstracts, animal studies, studies with adjuvant interventions and other non-research articles.
2. Trials that were not RCTs.
3. The subjects of the trials were not human participants.
4. The required outcome indicators were not reported in the literature.

### Search screening methods

1. First preliminary screening of literature: The results from the search strategy were independently reviewed by two researchers, who primarily screened the titles and abstracts. Non-clinical studies, non-randomized studies, and articles unrelated to the treatment of immune-mediated diseases with upadacitinib were excluded. They also excluded articles because of their types, subjects and other criteria set in advance.
2. Then full-text screening: A detailed review of the full text of the remaining articles was conducted based on the inclusion and exclusion criteria to determine the final studies included.
3. In case of disagreement between the two researchers during literature selection, the final decision was made through discussion with all researchers.

### Data extraction and quality assessment

The included RCTs were quantitatively assessed according to the Cochrane Risk of Bias Tools. For the possible sources of bias risk arising from improper experimental methods or the limitations of the sample itself in the research process, three evaluations are given: high risk, unclear risk, and low risk (11). Risk of bias graphs were generated using RevMan 5.3 or Stata 17.0 software.

Basic information and clinical data from the RCTs were manually extracted and recorded. If any necessary data were missing, attempts were made to contact the original authors. The above procedures were performed independently by two researchers.

### Statistical analysis

Study heterogeneity was assessed using the P-value from the chi-squared test and the inconsistency index (I^2^) (12). When heterogeneity was low (*P* > 0.05, I^2^ ≤ 50%), data were combined using a fixed-effects model. If the *P*-value was less than 0.05 or I^2^ exceeded 50%, significant heterogeneity was assumed. In such cases, the data were combined and analyzed using a random-effects model (13).

Dichotomous data were analyzed using risk ratio (RR) with 95% confidence intervals (95%CI). Continuous data were described as means and standard deviations (SD). Since some articles did not provide the means and SD directly, we had to estimate these values using available tools based on the sample sizes, 95%CI or other accessible data (14). Then Weighted mean differences (WMD) were used to analyze continuous variables with uniform measurement units. Continuous variables with non-uniform measurement units were analyzed by standardized mean differences (SMD). Statistical analysis data was all processed using RevMan 5.3 or Stata 17.0 software.

## Results

### Study selection and baseline characteristics

**Figure 1:**
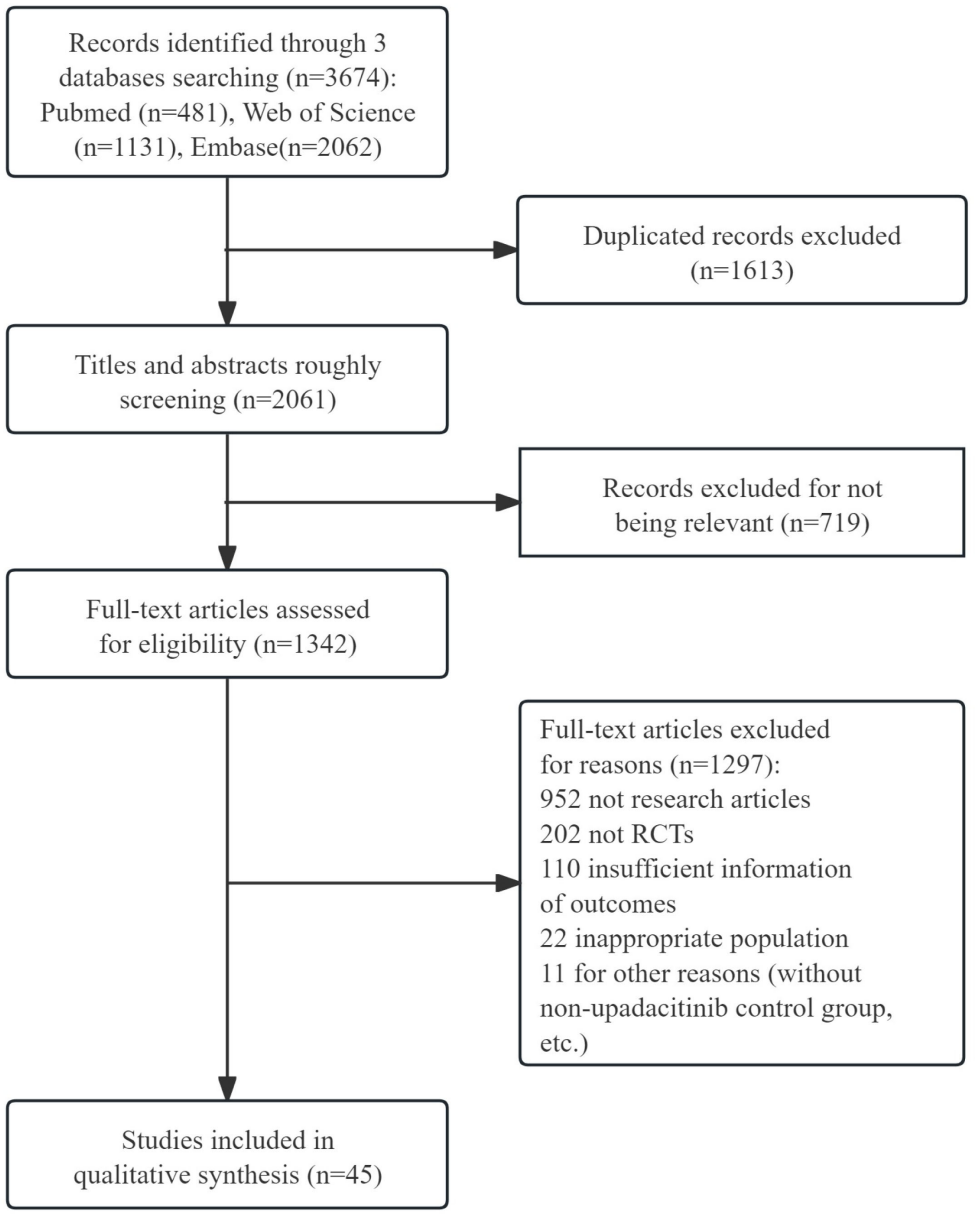
The flow diagram of literature researching and screening. RCTs, randomized controlled trials.

The literature searching and screening process is illustrated in **Figure 1**. We initially identified 3674 articles through relevant search terms. After removing 1,613 duplicates, we screened the remaining articles by title and abstract, resulting in the exclusion of 719 records. Following a full-text review, 1,297 records were excluded due to incomplete information or other reasons. Finally, 45 records (15–59) were included in the final meta-analysis. Key information such as author, publication year, disease type, subject characteristics (sample size), treatment details (upadacitinib dose, treatment duration), outcome indicators, and AEs were presented in **Supplementary Table S3**.

**Figure 2:**
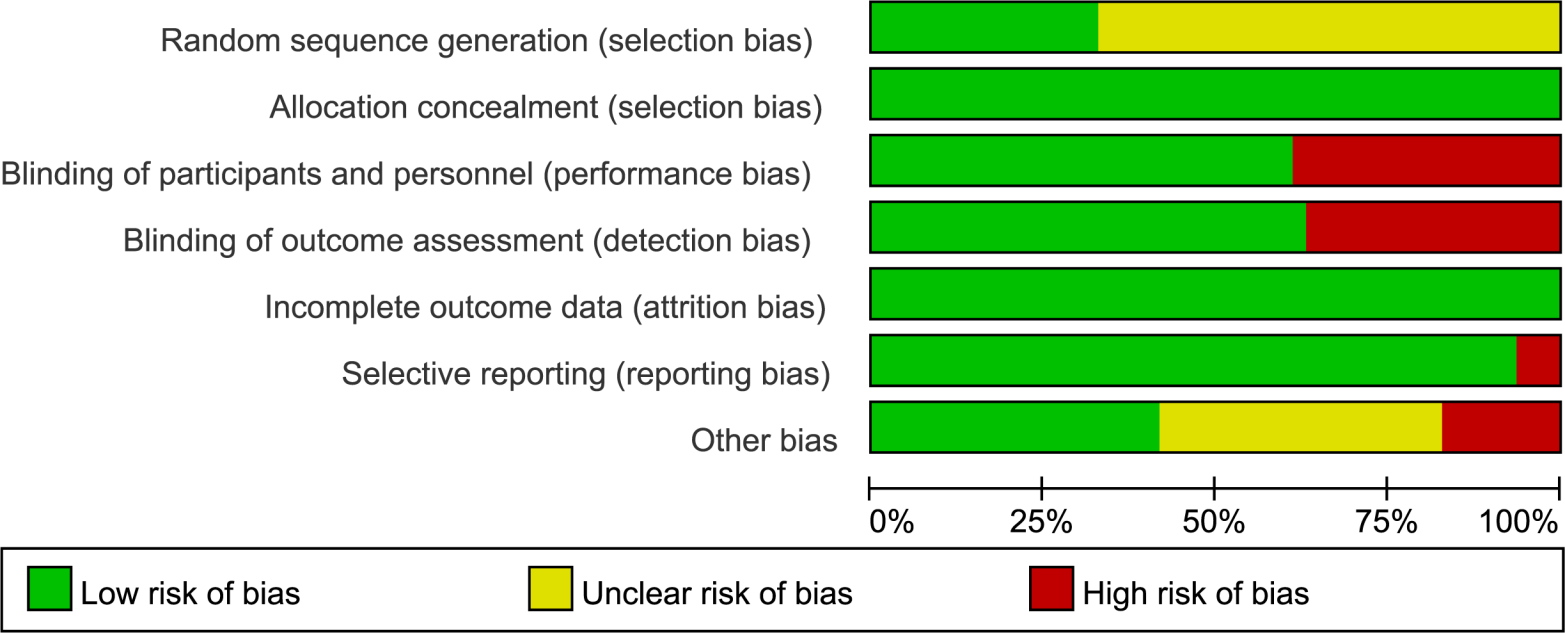
Risk of bias graph: review authors’ judgements about each risk of bias item presented as percentages across all included studies.

The graph of risk of bias was provided in **Figure 2** and the summary of the risk of bias was detailed in **Supplementary Figure S1**. The results noted the variability in the risk of bias across studies. For instance, several studies, particularly those with open-label designs, were assessed at a high risk. Several open-label studies were rated high risk, while some randomized double-blind studies lacked clear reporting of randomization and blinding, resulting in an unclear bias classification. These biases should be considered when interpreting the results, as they may impact the reliability and generalizability of the findings.

### Efficacy assessment

The efficacy outcomes across studies were summarized in **Supplementary Table S4**, with results stratified by disease type.

### Upadacitinib for RA

A total of 22 articles (16, 18, 19, 21, 23, 24, 26–30, 32, 33, 39, 42, 43, 49, 51–54, 59) were included in our study. At least 20% improvement according to ACR criteria (ACR20), 28-joint disease activity score using C-reactive protein [DAS28(CRP)] were used as primary endpoints. We also collected data on the clinical disease activity index (CDAI), simplified disease activity index (SDAI), 36-Item short form health survey (SF-36), and some other outcome indicators to comprehensively assess the efficacy of upadacitinib in treating RA.

**Figure 3:**
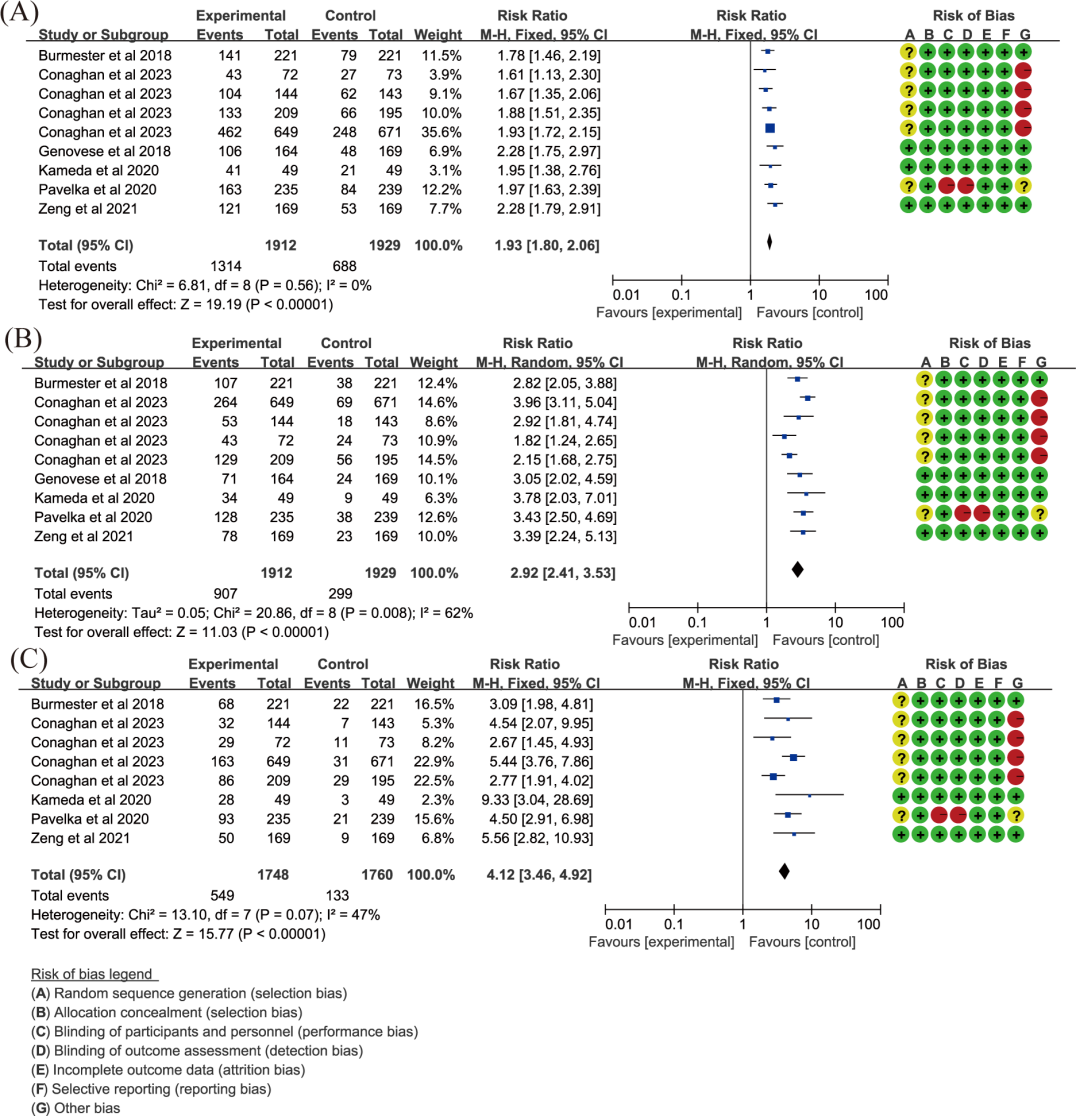
Key efficacy outcomes of upadacitinib 15 mg QD versus placebo for RA. **(A)** ACR20 **(B)** DAS28(CRP) LDA **(C)** DAS28(CRP) CR. Experimental: upadacitinib 15 mg QD; Control: placebo. The appearance of the same study is due to subgroup analyses or pooled analyses of different RCTs performed on the same experimental dose and control group with no duplication analyses. ACR20, at least 20% improvement in American College of Rheumatology Response Criteria; CR, clinical remission; DAS28(CRP), 28-joint Disease Activity Score using C-reactive Protein; LDA, low disease activity; QD, once daily; RA, rheumatoid arthritis.

Twelve essays (18, 19, 24, 26–30, 33, 42, 49, 59) reported ACR20. In comparison with placebo, upadacitinib 15 mg once daily (QD) improved ACR20 (RR = 1.93, 95%CI [1.80,2.06], *P* < 0.001) (**Figure 3A**), while 30 mg daily did not show a greater increase in efficacy (RR = 1.90, 95%CI [1.64,2.20], *P* < 0.001) (**Supplementary Figure S2A**). Fleischmann et al. and Pavelka et al. (26–29, 42) concentrated on the efficacy of upadacitinib compared to adalimumab (ADA), and the results demonstrated that upadacitinib 15 mg QD had more benefits than ADA 40 mg every other week (EOW) in achieving ACR20 (**Supplementary Figure S2B**).

Low diseases activity [LDA, DAS28(CRP) ≤ 3.2] was evaluated in the same 12 articles (18, 19, 24, 26–30, 33, 42, 49, 59). Clinical remission [CR, DAS28(CRP) < 2.6] was also included in 11 articles, excluding Genovese et al. (30). Other articles demonstrated that upadacitinib, at both 15 mg QD (**Figures 3B, C**) and 30 mg QD (**Supplementary Figures S2C, D**) doses, was more effective than placebo in achieving more patients reaching LDA and CR as measured by DAS28(CRP) (RR > 1, *P* < 0.05). Additionally, when compared to ADA, upadacitinib 15 mg QD also showed superiority over placebo (RR = 1.36, *P* < 0.001; **Supplementary Figures S2E, F**). However, it is worth noting that there was significant heterogeneity in analyses, and future research should focus on addressing this variability.

LDA and CR using CDAI or SDAI were also evaluated in our meta-analysis, indicating that upadacitinib had advantages over placebo or ADA in improving CDAI and SDAI (**Supplementary Table S4**; RR > 1, *P* < 0.001). However, the dose was not totally consistent with the efficacy in upadacitinib group. Concerning the assessment of daily quality of life, we included outcomes such as Health Assessment Questionnaire-Disability Index (HAQ-DI), pain relief, morning stiffness and SF-36. Compared to non-upadacitinib therapies, upadacitinib did not show superior effects in reducing pain (*P* > 0.05). Regarding morning stiffness, upadacitinib could reduce both the severity and the duration. However, upadacitinib 15 mg QD was similar to ADA 40 mg EOW in reducing the time of morning stiffness. In the SF-36 assessment, we mainly focused on the summary scores including physical component summary (PCS) and mental component summary (MCS). The results indicated that upadacitinib significantly improved SF-36 PCS. While, upadacitinib 30 mg QD did not show additional benefits compared to placebo, and its effect on improving SF-36 MCS was similar to that of ADA.

### Upadacitinib for axSpA

The primary endpoints in axSpA trials typically focus on improvements in spondyloarthritis international society response (ASAS), ASDAS, BASDAI, and others. Additional outcomes include reduction in back pain, changes in the bath ankylosing spondylitis functional index (BASFI), aspondyloarthritis research consortium of Canada (SPARCC) MRI Spine and sacroiliac joint inflammation scores, etc.

**Figure 4:**
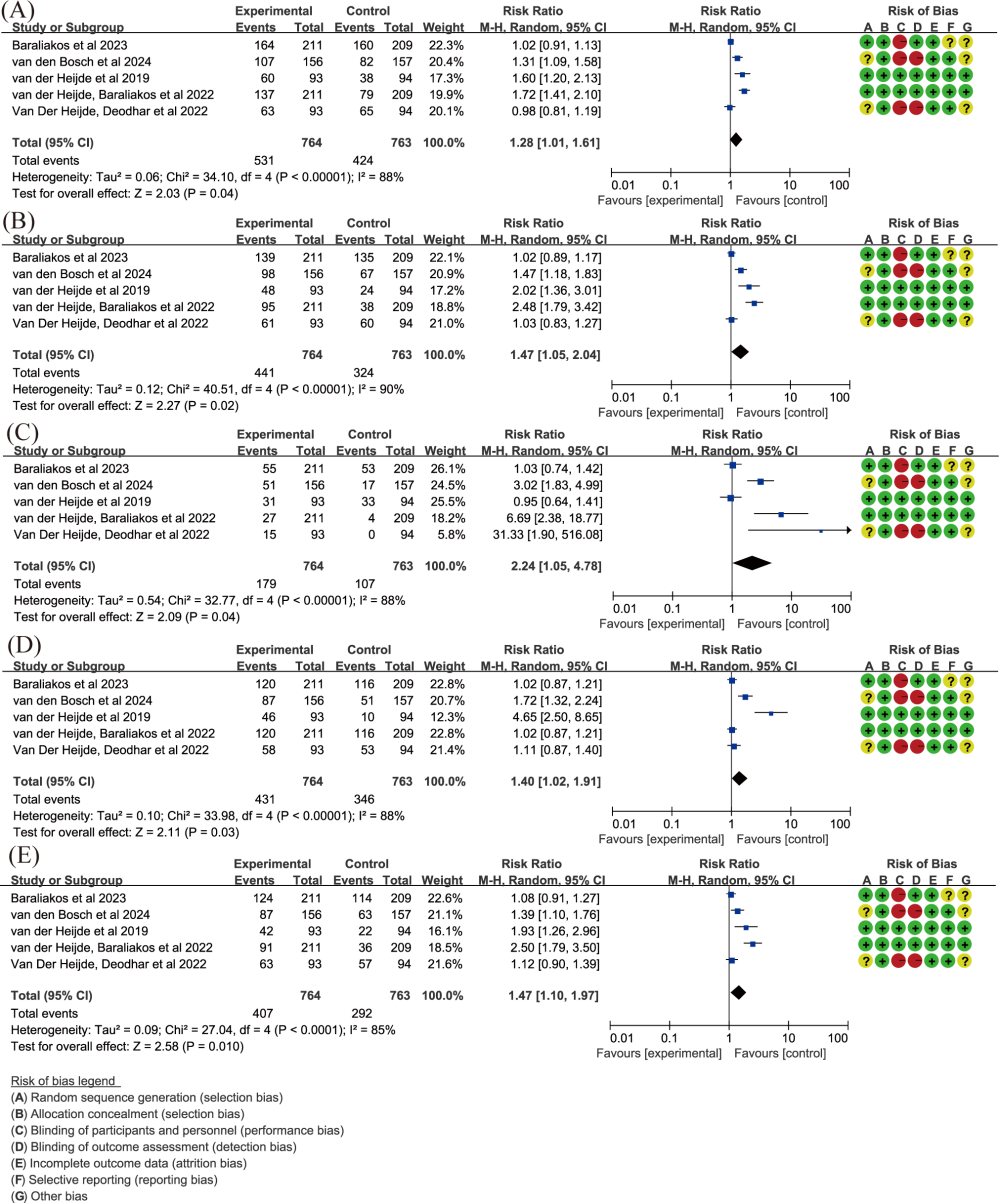
Key efficacy outcomes of upadacitinib 15 mg QD versus placebo for axSpA. **(A)** ASAS20 **(B)** ASAS40 **(C)** ASDAS ID **(D)** ASDAS LDA **(E)** BASDAI50. Experimental: upadacitinib 15 mg QD; Control: placebo. axSpA, axial spondyloarthritis; ASAS20/40, at least 20%/40% improvement in Assessment of SpondyloArthritis International Society; ASDAS, Ankylosing Spondylitis Disease Activity; BASDAI50, at least 50% improvement in Bath Ankylosing Spondylitis Disease Activity Index; ID, inactive disease; LDA, low disease activity; QD, once daily.

Regarding ASAS, due to high heterogeneity (*P* ≤ 0.05, I^2^ > 50%), random-effects models were used. The results from five articles (15, 55–58) demonstrated that upadacitinib 15 mg QD led to greater improvements in ASAS, with RR for ASAS20 and ASAS40 being 1.28 and 1.47 respectively (**Figures 4A, B**). ASDAS, another important marker for assessing disease severity in spondyloarthritis, was analyzed in our study. The same five articles (15, 55–58), using random-effects models due to heterogeneity, with statistically significant improvements (*P* < 0.05) observed for both ASDAS inactive disease (ID) and LDA (**Figures 4C, D**). In terms of improvement of over 50% in BASDAI (BASDAI50), upadacitinib 15 mg QD also showed superiority over placebo (RR = 1.47, 95%CI [1.10, 1.97]) (**Figure 4E**).

Spinal arthropathy often causes back pain, and upadacitinib significantly alleviated total back pain (*P* = 0.04), although it did not significantly improve nocturnal back pain (*P* = 0.09). Additionally, in terms of functional impairment as measured by BASFI, upadacitinib led to greater improvements compared to placebo, with a significant difference (WMD = -0.7, 95% CI [-1.18, -0.22], *P* = 0.004).

### Upadacitinib for PsA

**Figure 5:**
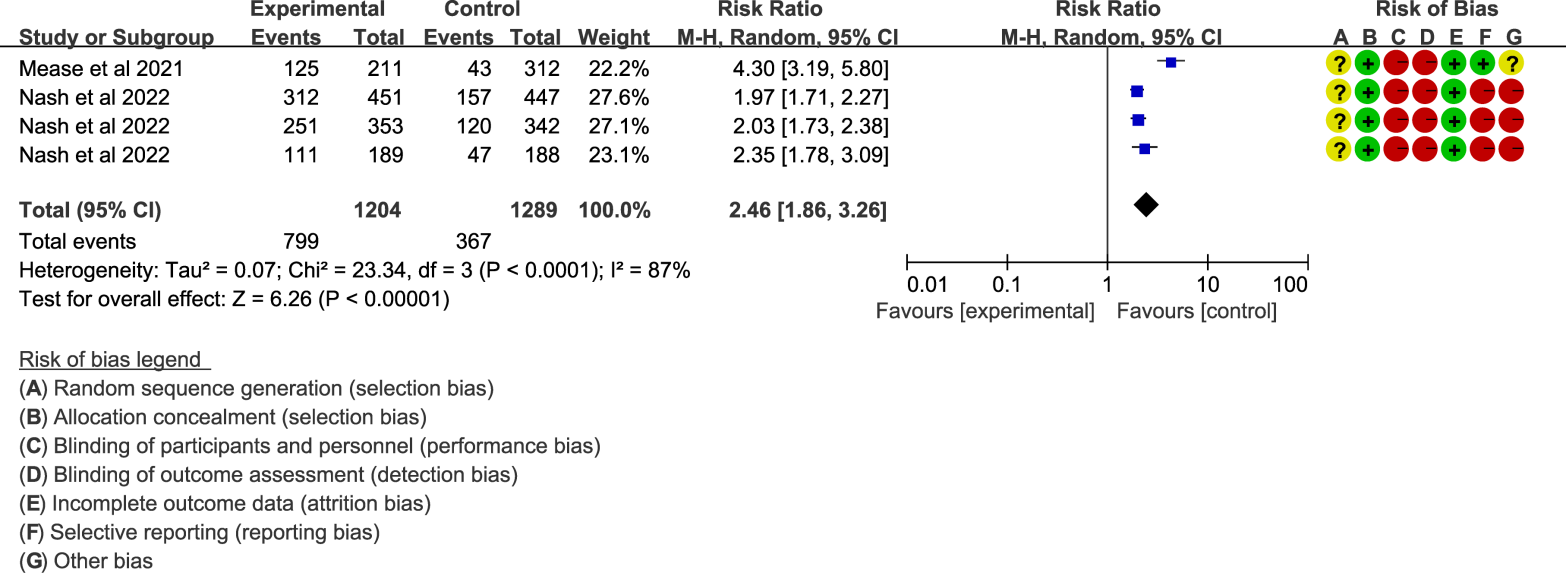
ACR20 of upadacitinib 15 mg QD versus placebo for PsA. Experimental: upadacitinib 15 mg QD; Control: placebo. The appearance of the same study is due to subgroup analyses or pooled analyses of different RCTs performed on the same experimental dose and control group with no duplication analyses. ACR20, at least 20% improvement in American College of Rheumatology Response Criteria; PsA, Psoriatic Arthritis; QD, once daily.

In PsA, ACR20 was a key endpoint, but high heterogeneity was observed across the included trials (38, 40) (I^2^ > 50%, *P* < 0.05). Results showed that upadacitinib 15 mg QD (**Figure 5**) or 30 mg QD (**Supplementary Figure S3**) led to a higher proportion of patients achieving ACR responses compared to placebo (RR = 2.46/2.68, *P* < 0.001). The ACR70/100 assessments also favored upadacitinib (RR > 1, *P* < 0.001).

In addition to primary outcomes, several secondary outcomes were also assessed. Regarding psoriasis severity, improvements in PASI scores (PASI75/90/100) demonstrated that upadacitinib was superior to placebo (RR > 1, *P* < 0.001). Regarding complications of PsA, such as dactylitis and enthesitis, upadacitinib showed significant improvement in enthesitis, but did not show significant improvement in dactylitis, based on the Leeds Enthesitis Index (LEI = 0) and Leeds Dactylitis Index (LDI = 0). For the quality of life, as measured by the HAQ-DI in four articles (36, 38, 40, 50), upadacitinib 15 mg or 30 mg QD outperformed both placebo and ADA 40 mg EOW.

### Upadacitinib for CD

**Figure 6:**
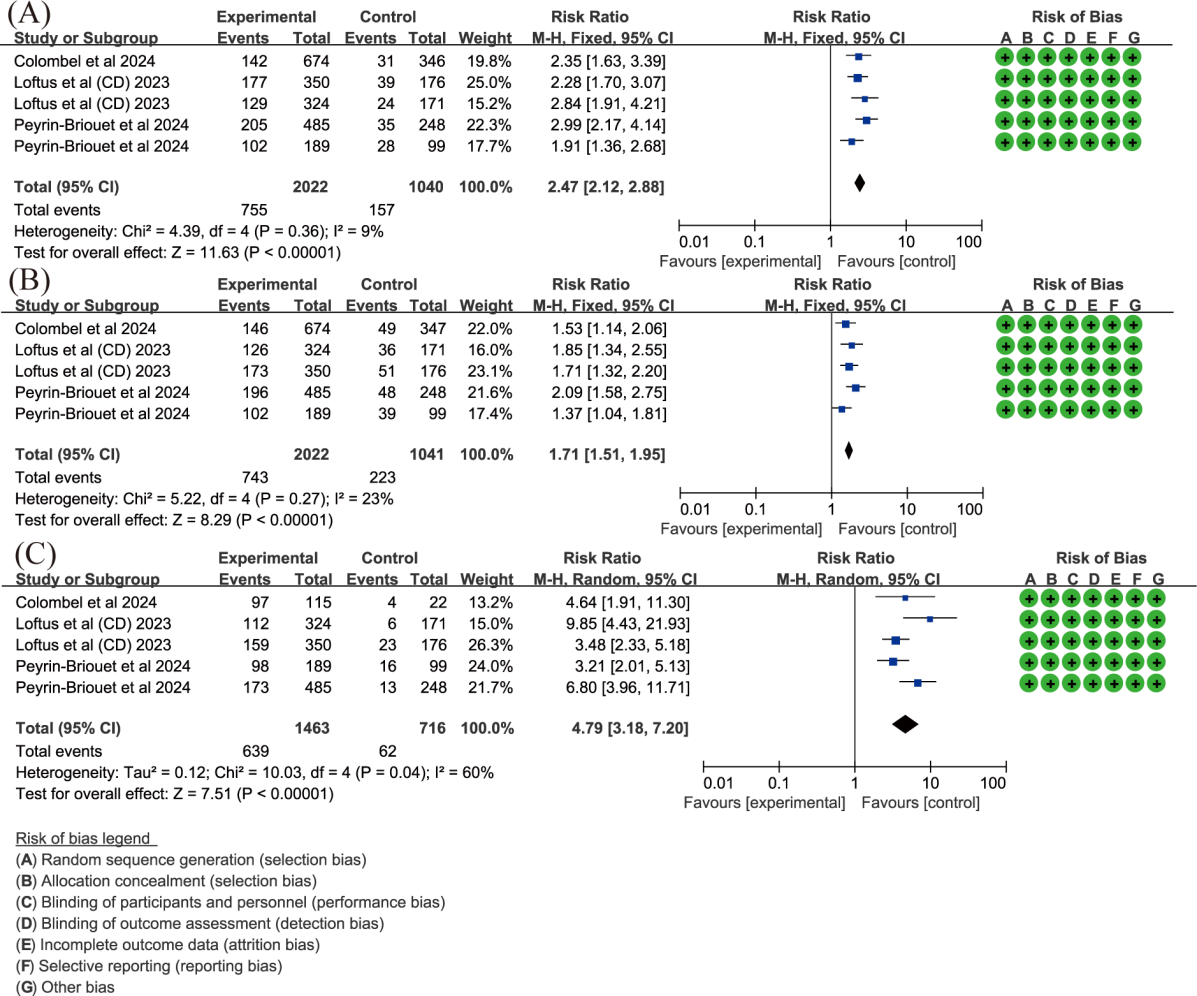
Key efficacy outcomes of upadacitinib 45 mg QD versus placebo for CD. **(A)** SF/APS CR **(B)** CDAI CR **(C)** endoscopic response. Experimental: upadacitinib 45 mg QD; Control: placebo. The appearance of the same study is due to subgroup analyses or pooled analyses of different RCTs performed on the same experimental dose and control group with no duplication analyses. APS, Abdominal Pain Score; CR, clinical remission; CD, Crohn’s Disease; CDAI, Crohn’s Disease Activity Index; QD, once daily; SF, Stool Frequency.

In the meta-analysis for CD, key endpoints such as Stool Frequency/Abdominal Pain Score (SF/APS) CR, Crohn’s Disease Activity Index (CDAI) CR, and endoscopic response were evaluated in three records (22, 35, 45). SF/APS CR is defined by average daily very soft/liquid SF ≤ 2.8 and an APS ≤ 1.0, with neither worsening from baseline, in patients with a baseline SF ≥ 4.0 or APS ≥ 2.0. A fixed-effects model was used with low heterogeneity (I^2^ = 9%, *P* = 0.36). The results showed that upadacitinib 45 mg QD resulted in a significantly higher proportion of patients achieving SF/APS clinical remission compared to placebo (RR = 2.47, 95%CI [2.12, 2.88], *P* < 0.001) (**Figure 6A**). A similar result was observed for CDAI CR, defined as CDAI < 150, with low heterogeneity (I² = 23%, *P* = 0.27). Upadacitinib 45 mg QD also showed a statistically significant improvement in CDAI clinical remission compared to placebo (RR = 1.71, 95% CI [1.51, 1.95], *P* < 0.001) (**Figure 6B**). For endoscopic response, defined as a more than 50% reduction in the simplified endoscopic score for CD (SES-CD) from baseline, a random-effects model was used due to moderate heterogeneity (I^2^ = 60%, *P* = 0.04). The results demonstrated that the proportion of patients achieving endoscopic response was significantly higher in the upadacitinib 45 mg QD group compared to the control group [RR = 4.79, 95%CI [3.18, 7.20]; *P* < 0.001] (**Figure 6C**).

The 15 mg and 30 mg QD doses also showed improvements in all three efficacy outcomes, although two articles (44, 47) were excluded from the analysis due to dose discrepancies. Nevertheless, both studies suggested upadacitinib’s potential for achieving CR and endoscopic response, with Peyrin-Biroulet et al. (44) noting improvements in health-related quality of life (HRQOL) and work productivity with upadacitinib treatment.

### Upadacitinib for UC

**Figure 7:**
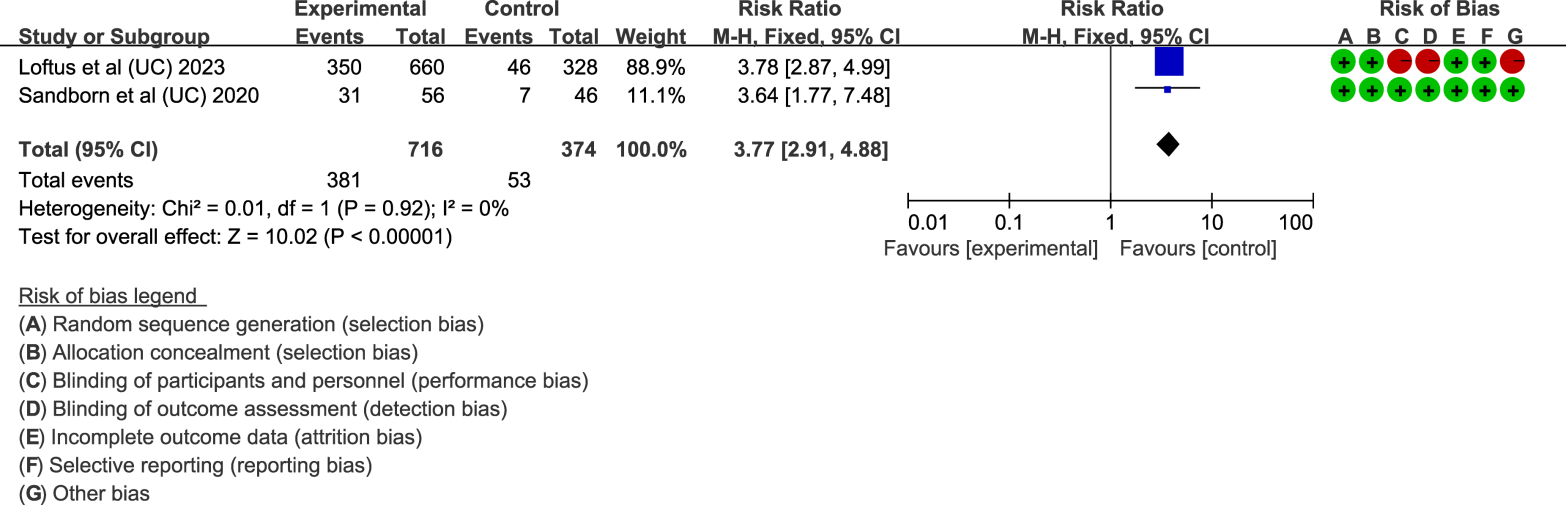
Clinical remission rates of upadacitinib 45 mg QD versus placebo for UC. UC, ulcerative colitis; QD, once daily.

In UC, CR is also a key endpoint. Two articles (34, 48) assessed the proportion of patients achieving CR with upadacitinib 45 mg QD. The results, with low heterogeneity (I² < 50%, *P* > 0.05), showed that upadacitinib significantly outperformed placebo in achieving CR, with a higher proportion of patients achieving remission (RR = 6.92, 95%CI [4.99, 9.59]; *P* < 0.001) (**Figure 7**).

Several secondary endpoints were also evaluated, including stool frequency score (SFS), rectal bleeding score (RBS), abdominal pain score (APS) and bowel urgency. Three essays (31, 34, 48) focused on improvements in stool conditions, specifically the proportion of patients achieving an RBS of 0 or an SFS < 1. Fixed-effects models (I² < 50%, *P* > 0.05) showed that upadacitinib was superior to placebo (*P* < 0.001) in these outcomes. For abdominal pain and bowel urgency, Danese et al., Ghosh et al., and Loftus et al. (25, 31, 34) found significant improvements in upadacitinib 45 mg QD versus placebo, with a *P*-value < 0.05 for both outcomes. Furthermore, three studies investigated the impact of upadacitinib on quality of life. Ghosh et al. and Loftus et al. (31, 34) reported on the IBD questionnaire (IBDQ) response, defined as an increase of at least 16 points from baseline. Both studies found that upadacitinib-treated patients were more likely to achieve a positive IBDQ response. Panés et al. (41) also demonstrated that upadacitinib improved HRQOL measures both during the induction phase with 45 mg and the maintenance phase with 15 mg or 30 mg.

### Safety assessment

The safety profiles were described based on the reported frequency of AEs from twenty-nine articles (15, 17–21, 26–30, 32, 33, 35, 36, 38–40, 42, 45–49, 55–59). Of these studies, Baraliakos et al. and Van Der Heijde, Deodhar et al. only conducted a described statistics and indicated that no new safety findings were observed with upadacitinib treated AS patients (15, 57).

**Figure 8:**
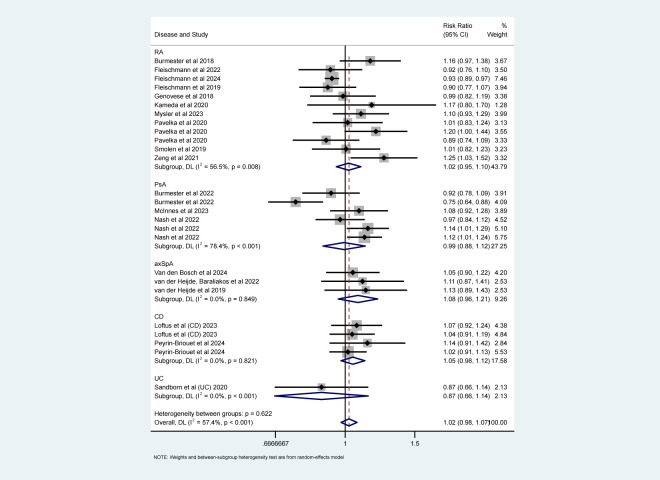
Safety analyses (overall AEs) about upadacitinib of recommended doses versus non-upadacitinib therapies. The appearance of the same study is due to subgroup analyses or pooled analyses of different RCTs performed on the same experimental dose and control group with no duplication analyses. axSpA, Axial Spondyloarthritis; CD, Crohn’s disease; PsA, psoriatic arthritis; RA, rheumatoid arthritis; UC, ulcerative colitis.

The results of overall AEs, categorized by the recommended dose for each specific disease, are shown in **Figure 8**. The analysis indicated that upadacitinib’ s safety profile was comparable to non-upadacitinib therapies (RR = 1.02, 95% CI [0.98, 1.07]).

**Figure 9:**
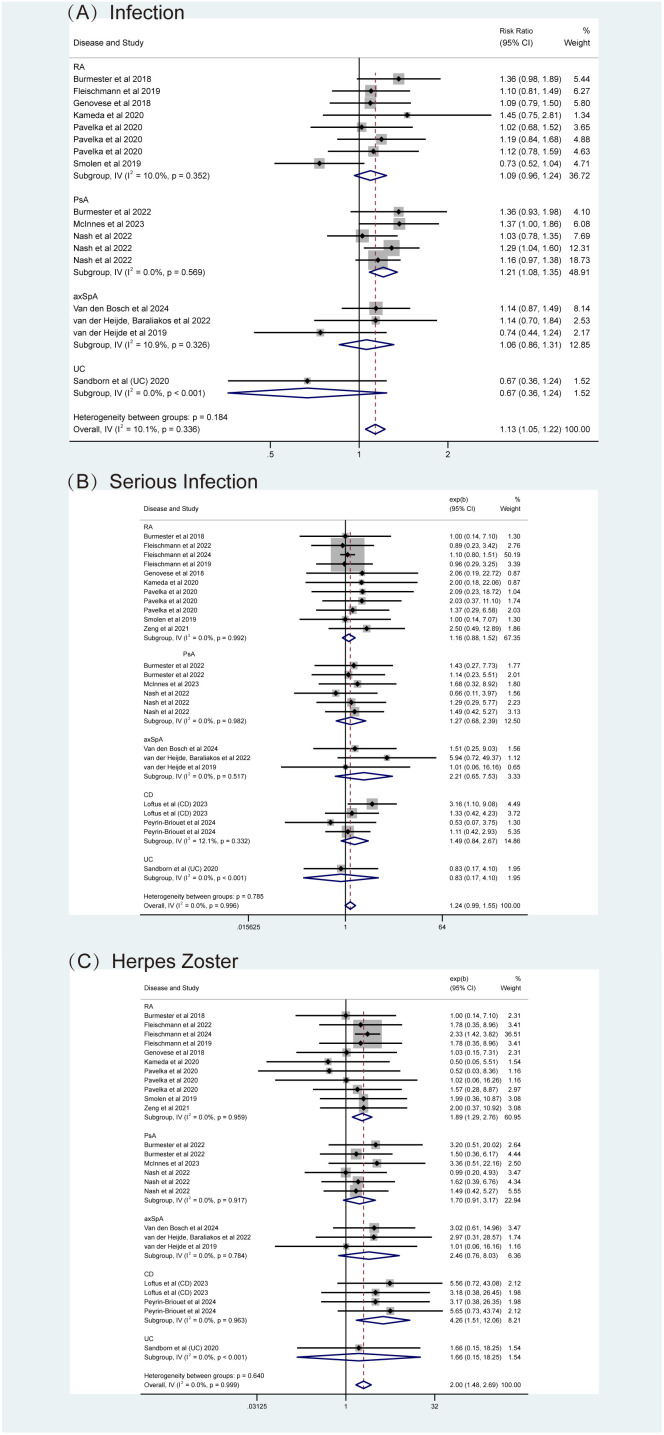
Adverse events (AEs) related to infections for upadacitinib at recommended doses versus non-upadacitinib therapies. **(A)** Infections, **(B)** Serious Infections, **(C)** Herpes Zoster. The appearance of the same study across different plots is due to subgroup or pooled analyses of various RCTs conducted with the same experimental dose and control group, ensuring no duplication of analyses. axSpA, Axial Spondyloarthritis; CD, Crohn’s disease; PsA, psoriatic arthritis; RA, rheumatoid arthritis; UC, ulcerative colitis.

Specific AEs were analyzed, including serious adverse events (SAEs), death, infections (overall and serious), herpes zoster (HZ), malignancy excluding non-melanoma skin cancer, (NMSC), NMSC, major adverse cardiovascular events (MACE), and venous thromboembolism events (VTE). Fixed-effects models (I² < 50%, *P* > 0.05) showed that upadacitinib was associated with a slight increase in the risk of infection (RR = 1.13, 95% CI [1.05, 1.22]), particularly with a higher risk of HZ (RR = 2.00, 95% CI [1.48, 2.69]) (**Figures 9A, C**). Although there was a higher incidence of serious infections (RR = 1.24), the difference was not statistically significant (**Figure 9B**). Furthermore, no new safety concerns were observed with respect to SAEs, death, malignancy, NMSC, MACE, or VTE, as the 95% confidence intervals crossed 1 (**Supplementary Figure S4**). Additionally, some studies indicated that upadacitinib might increase the risk of elevated creatine kinase (CPK) and dyslipidemia.

Subgroup analyses by types of IMIDs also revealed important findings. As shown in **Figures 8**, **9** and **Supplementary Figure S4**, upadacitinib did not significantly increase the overall risk of AEs across five diseases, with RRs were approximately equal to 1 and 95% CIs consistently including 1. In RA, upadacitinib showed a similar safety profile compared to placebo, ADA, and MTX, except for a higher risk of HZ (RR = 1.89, 95% CI [1.29, 2.70]). In PsA, there were no significant differences in specific AEs between upadacitinib and non-upadacitinib treatment groups, though upadacitinib was associated with a higher risk of infections (RR = 1.21, 95% CI [1.08, 1.35]). In axSpA and CD patients, no significant differences in safety assessment compared to non-upadacitinib groups were found, with 95% CI crossing 1 for all included AEs. Regarding UC, only one study by Sandborn et al. (48) reported AEs, showing that the incidence of AEs was similar across all upadacitinib dose groups (7.5 mg, 15 mg, 30 mg, 45 mg QD: 63.8%, 61.2%, 69.2%, 62.5%, respectively), and slightly higher in the placebo group (71.7%). This study also identified several risk factors for increased likelihood of AEs, such as age over 65, smoking history, hospitalization, fluid loss, bed rest during UC flare-ups, and concurrent corticosteroid use, etc.

Regarding dose-dependent safety effects, Sandborn et al. (47) investigated different doses of upadacitinib and found that the incidence of AEs increased with higher doses. Our analysis of available data confirmed that more AEs in total were reported in the upadacitinib 30 mg group compared to the 15 mg group (RR = 0.85, 95% CI [0.82, 0.89]; **Supplementary Figure S5**).

## Discussion

Upadacitinib, a selective JAK1 inhibitor, has demonstrated significant efficacy in treating a variety of immune-mediated diseases, including AD, RA, axSpA, PsA, UC and CD. To our knowledge, this is the first systematic review to comprehensively evaluate the safety and efficacy of upadacitinib across such a broad spectrum of IMIDs.

Our findings confirm the efficacy of upadacitinib in multiple immune-mediated diseases. In RA and PsA patients, upadacitinib significantly improved disease activity compared to placebo, with the RR greater than 1 and the *P-value* < 0.05, underscoring its therapeutic potential. However, when compared to ADA, the results were less definitive. As shown in **Supplementary Table S4**, the 95% CIs for key outcomes included 1, and the *P-values* exceeded 0.5. For instance, in RA patients, the improvement in morning stiffness duration was not significantly different from ADA (SMD = -8.15, 95% CI [-20.33, 4.03]), suggesting comparable efficacy between the two treatments in this regard. Moreover, while upadacitinib demonstrated benefits in disease activity, its impact on pain relief appeared more limited. In RA, upadacitinib did not significantly outperform placebo in alleviating pain (*P* = 0.28). Similarly, in PsA, upadacitinib 15 mg and 30 mg QD did not show significant advantages over ADA in terms of pain relief, with SMDs of -0.41 (95% CI [-0.82, 0.01]) and -0.30 (95% CI [-0.69, 0.08]), respectively (*P* > 0.05). Additionally, upadacitinib 15 mg did not show significant advantages over ADA 40 mg EOW in alleviating rash, as assessed by PASI (RR ≤ 1). In axSpA patients, upadacitinib demonstrated advantages in reducing disease activity. It was effective in reducing overall pain (SMD = -0.80, 95%CI [-1.56,-0.05]), though it did not significantly alleviate nocturnal back pain (SMD = -0.72, 95%CI [-1.55,0.11]). These findings suggest that while upadacitinib is effective for managing disease activity, its benefits for symptom management remain modest. Further studies are needed to optimize its role in symptom management, potentially through dose adjustments or combination therapies.

Additionally, the accumulated evidence indicates that upadacitinib may provide significant relief for arthritis by reducing structural damage. Secondary outcomes, such as changes in the modified total Sharp/van der Heijde score (△mTSS ≤ 0), have already been assessed and can be found in **Supplementary Table S4**. Across RA trials (24, 26, 27, 29, 42, 43), upadacitinib demonstrated a protective effect on joint structure compared to placebo (evaluated by △mTSS ≤ 0, RR = 1.14, 95% CI [1.11, 1.18]), although this effect was similar to that of ADA, with a *P-value* of 0.72. Besides, the efficacy of upadacitinib appeared to be independent of concomitant MTX use, suggesting the potential for this drug to be an option for patients who are intolerant to MTX. For PsA, consistent inhibition of radiographic progression was observed with upadacitinib (36). In axSpA, significant improvements in the SPARCC MRI spine or sacroiliac joint scores (SMDs both less than 0, *P-values* both 0.002) (56–58) imply a unique capacity to target both axial and peripheral joint pathology - a distinction from TNF inhibitors’ more limited axial effects. These findings underline the potential of upadacitinib in preventing joint damage progression, a critical consideration in managing long-term outcomes of inflammatory arthritis. Future research should aim to further delineate upadacitinib’ s precise role in halting or reversing structural damage, especially in different arthritis subtypes, to better guide clinical decision-making and optimize treatment strategies.

Upadacitinib is currently the only one JAK inhibitor approved for the treatment of IBD. Our study provided robust evidence that upadacitinib enabled faster clinical remission and endoscopic response and significantly alleviated symptoms such as abdominal pain, diarrhea, and rectal bleeding. In addition, the drug is associated with a rapid onset of action, with some patients experiencing significant symptom relief within a few days or weeks of starting treatment (22, 34). However, the included studies only compared upadacitinib with placebo. Further trials comparing upadacitinib to other treatment (e.g., biologics) are required, particularly in well-defined patient populations, to better assess its relative efficacy.

Regarding safety, upadacitinib was generally well tolerated. Notably, there were no new risks of death or SAEs with upadacitinib treatment across multiple IMIDs (**Figures 8**, **9**; **Supplementary Figure S4**). However, specific safety concerns were identified. Our analysis found that upadacitinib was associated with an increased risk of infections, particularly HZ, consistent with the known side effects of JAK inhibitors (20, 60, 61). In patients with compromised immune function, the use of upadacitinib requires careful management, and proactive measures (such as adequate rest, regular exercise, and close monitoring) should be taken to minimize these risks. Early administration of immune-enhancing agents, when appropriate, could further mitigate the risk of infections. NMSC has been observed in RA patients treated with upadacitinib, with a dose-dependent incidence (46). In PsA and axSpA studies, NMSC was seldom observed, and the meta-analysis results showed no significant difference between upadacitinib and control groups. Malignancy excluding NMSC was also evaluated and our findings were consistent with the *post-hoc* pooled analyses (17, 46). As a selective JAK1 inhibitor, upadacitinib affects T and NK cells, which are critical for immune surveillance and cancer detection. This raises concerns about the potential impact on immune surveillance and subsequent cancer risk (62). Nevertheless, the incidence of NMSC and malignancy remains low, regular checks are critical for patients at risk. Other long-term safety concerns, such as MACE and VTE, have been raised by scholars, but no new risk signals were identified in our safety assessment (15, 38). Although rare, these concerns underscore the importance of pre-treatment screening and careful monitoring, particularly for patients with known risk factors such as advanced age and obesity. Prolonged observation is essential for the assessment and management of chronic AEs, including tumors, MACE, and VTE.

Laboratory indicators monitoring is critical in clinical trials involving upadacitinib. In addition to inflammatory markers reflecting disease activity, common tests include blood routine, liver enzymes, lipid status, CPK levels and other hematological parameters. Hematological changes such as anemia, lymphopenia, neutropenia, and thrombocytopenia have also been reported, though these are generally mild and resolve with symptomatic treatment or drug discontinuation. Given that upadacitinib selectively targets JAK1 without affecting JAK2 and JAK3, which are both critical for erythropoiesis and immune function, it carries a lower risk of hematological abnormalities compared to non-selective JAK inhibitors. Nonetheless, regular blood tests remain critical to detect abnormalities early and minimize risks. Furthermore, no clear association between upadacitinib and drug-induced liver injury has been observed. Patients receiving upadacitinib in combination with MTX were more likely to experience increases in liver enzymes (21). Elevated CPK levels were common but usually asymptomatic (20, 40, 58). JAK1 activation phosphorylates STAT3, which plays a role in skeletal muscle activation and may contribute to CPK elevation. Interestingly, Queeney et al. (63) suggested that CPK elevation may reflect recovery from inflammation-induced muscle inhibition rather than muscle injury. Given that JAK inhibitors may cause an increase in CPK, regular monitoring and attention to muscle symptoms are particularly important. Blood lipid elevations have been observed in several studies (21, 60). However, the relationship between dyslipidemia and the risk of cardiovascular events in patients receiving upadacitinib warrants further exploration.

In our study, we also conducted dose-specific analyses. Most findings suggested that higher doses of upadacitinib may offer greater benefits in symptoms alleviation. However, this potential benefit comes with a well-documented increase in the risk of AEs. Our analysis showed that more AEs were reported in the upadacitinib 30 mg group compared to the 15 mg group (RR = 0.85, 95% CI [0.82, 0.89]). Given these findings, a personalized treatment approach is necessary, where clinicians must carefully assess individual disease activity and tailor the dose accordingly to balance efficacy and safety.

Another point to note is that not all 45 records were included in our meta-analysis. For example, some studies used MTX or abatacept as the control group, and only one study reported results that could not be included in the meta-analysis but were presented in the efficacy outcomes summary (**Supplementary Table S4**). For RA, Smolen et al. (49) compared upadacitinib with MTX and found that upadacitinib was superior in achieving ACR20/50/70 responses, reducing DAS28(CRP) scores, and improving other outcomes. Similarly, Bergman et al. (16) used abatacept as the control group and demonstrated that upadacitinib outperformed abatacept in improving quality of daily life. Additionally, several studies included in this study focused on specific outcomes, such as laboratory biomarkers and imaging evaluations, which were not incorporated into the meta-analysis (21, 43). Nonetheless, these studies consistently supported the efficacy of upadacitinib and affirmed its acceptable safety.

Though our meta-analysis provides robust evidence for the efficacy and safety of upadacitinib, several limitations should be addressed, and several directions for future research remain. First, the significant heterogeneity observed across the included RCTs can largely be attributed to multiple factors, with variability in treatment duration emerging as one of the most influential. The studies in this analysis spanned a wide range of follow-up periods, from 8 to 264 weeks. This divergence in follow-up duration likely contributed to the observed variability in treatment outcomes, as shorter studies may not adequately capture the long-term effects of upadacitinib, including sustained therapeutic benefits, delayed adverse effects, or any shifts in treatment response over time. Additionally, baseline patient characteristics (e.g., disease severity, comorbidities) may contribute to variability in outcomes. Future research should aim for longer follow-up periods and consistent study designs to better assess the long-term impact and minimize these sources of variability. Furthermore, the limited number of high-quality RCTs and the potential bias (e.g., performance and detection bias in open-label studies) may impact the reliability and generalizability of our findings. For instance, the study by Nash et al. (40) was rated as having a high risk of bias primarily due to this issue. In such open-label designs, patient awareness of their treatment could lead to subjective outcome reporting, potentially skewing the results. While some studies were randomized and double-blind, the lack of detailed descriptions of randomization and blinding methods led to an ‘unclear’ risk of bias classification. Future studies should involve larger, well-conducted RCTs with proper randomization, blinding, and clear inclusion/exclusion criteria. Real-world data from observational studies and post-marketing surveillance will also be crucial in evaluating long-term safety and efficacy. Lastly, future research should focus on comparing upadacitinib with other biologics or traditional therapies in head-to-head studies to assess its relative efficacy. Furthermore, more research is needed to refine dosing strategies based on specific diseases, patient populations, and overlapping conditions. Understanding how upadacitinib affects the downstream cytokine pathways and identifying risk factors influencing its efficacy will be critical for personalizing treatment. In clinical practice, a comprehensive assessment of the patients’ status is critical for optimizing both therapeutic efficacy and minimizing risks. In the future, exploring the potential of upadacitinib in treating other off-label immune-related diseases, such as SS, SLE and LN, dermatomyositis, could further expand its therapeutic applications. This would further solidify upadacitinib’ position as a versatile and promising treatment option in the immune-mediated disease landscape.

## Conclusion

Overall, the great therapeutic potential of upadacitinib is clear, demonstrating substantial efficacy across a range of IMIDs. It effectively alleviates symptoms, reduces disease activity, and shows notable benefits in improving quality of life. Due to the heterogeneity in our research, the real-world benefits of upadacitinib may vary significantly based on individual patient factors, and further research is needed to clarify its impact on symptoms, quality of life, and overall efficacy. Additionally, the safety profile is generally manageable, but careful monitoring for risks such as infections especially HZ is necessary. A personalized treatment approach (including medicine time, dose, frequency, etc.), considering both efficacy and safety, is crucial to optimizing outcomes for individual patients.

## Data Availability

The original contributions presented in the study are included in the article/**Supplementary Material**. Further inquiries can be directed to the corresponding authors.
