# Supplementary material for: Efficacy and safety of upadacitinib for patients with immune-mediated inflammatory diseases: a systematic review and meta-analysis
Source: Front Immunol. 2025 Jul 1;16:1586792. doi: 10.3389/fimmu.2025.1586792 (PMC12259590; doi:10.3389/fimmu.2025.1586792)
Supplement: Supplementary file 1 [file DataSheet1.pdf]

Additional file 1

Table S1. PRISMA 2020 Checklist.

Table S2. Search Strategies.

Table S3. Main characteristics of the included studies.

Table S4. Efficacy Outcomes Summary: Upadacitinib vs Non-Upadacitinib in RA, axSpA, PsA, CD, and UC.

Table S1.PRISMA 2020 Checklist.			
**Section and Topic**	**Item #**	**Checklist item**	**Location where item is reported**
**TITLE**			
Title	1	Identify the report as a systematic review.	1
**ABSTRACT**			
Abstract	2	See the PRISMA 2020 for Abstracts checklist.	1-2
**INTRODUCTION**			
Rationale	3	Describe the rationale for the review in the context of existing knowledge.	2-4
Objectives	4	Provide an explicit statement of the objective(s) or question(s) the review addresses.	2-4
**METHODS**			
Eligibility criteria	5	Specify the inclusion and exclusion criteria for the review and how studies were grouped for the syntheses.	4-6
Information sources	6	Specify all databases, registers, websites, organisations, reference lists and other sources searched or consulted to identify studies. Specify the date when each source was last searched or consulted.	4-6
Search strategy	7	Present the full search strategies for all databases, registers and websites, including any filters and limits used.	4-6
Selection process	8	Specify the methods used to decide whether a study met the inclusion criteria of the review, including how many reviewers screened each record and each report retrieved, whether they worked independently, and if applicable, details of automation tools used in the process.	4-6
Data collection process	9	Specify the methods used to collect data from reports, including how many reviewers collected data from each report, whether they worked independently, any processes for obtaining or confirming data from study investigators, and if applicable, details of automation tools used in the process.	4-6
Data items	10a	List and define all outcomes for which data were sought. Specify whether all results that were compatible with each outcome domain in each study were sought (e.g. for all measures, time points, analyses), and if not, the methods used to decide which results to collect.	4-6
	10b	List and define all other variables for which data were sought (e.g. participant and intervention characteristics, funding sources). Describe any assumptions made about any missing or unclear information.	4-6
Study risk of bias assessment	11	Specify the methods used to assess risk of bias in the included studies, including details of the tool(s) used, how many reviewers assessed each study and whether they worked independently, and if applicable, details of automation tools used in the process.	4-6
Effect measures	12	Specify for each outcome the effect measure(s) (e.g. risk ratio, mean difference) used in the synthesis or presentation of results.	4-6
Synthesis methods	13a	Describe the processes used to decide which studies were eligible for each synthesis (e.g. tabulating the study intervention characteristics and comparing against the planned groups for each synthesis (item #5)).	4-6
	13b	Describe any methods required to prepare the data for presentation or synthesis, such as handling of missing summary statistics, or data conversions.	4-6
	13c	Describe any methods used to tabulate or visually display results of individual studies and syntheses.	4-6

Table S1.PRISMA 2020 Checklist.			
**Section and Topic**	**Item #**	**Checklist item**	**Location where item is reported**
	13d	Describe any methods used to synthesize results and provide a rationale for the choice(s). If meta-analysis was performed, describe the model(s), method(s) to identify the presence and extent of statistical heterogeneity, and software package(s) used.	4-6
	13e	Describe any methods used to explore possible causes of heterogeneity among study results (e.g. subgroup analysis, meta-regression).	4-6
	13f	Describe any sensitivity analyses conducted to assess robustness of the synthesized results.	4-6
Reporting bias assessment	14	Describe any methods used to assess risk of bias due to missing results in a synthesis (arising from reporting biases).	4-6
Certainty assessment	15	Describe any methods used to assess certainty (or confidence) in the body of evidence for an outcome.	4-6
**RESULTS**			
Study selection	16a	Describe the results of the search and selection process, from the number of records identified in the search to the number of studies included in the review, ideally using a flow diagram.	6-48
	16b	Cite studies that might appear to meet the inclusion criteria, but which were excluded, and explain why they were excluded.	6-48
Study characteristics	17	Cite each included study and present its characteristics.	6-48
Risk of bias in studies	18	Present assessments of risk of bias for each included study.	6-48
Results of individual studies	19	For all outcomes, present, for each study: (a) summary statistics for each group (where appropriate) and (b) an effect estimate and its precision (e.g. confidence/credible interval), ideally using structured tables or plots.	6-48
Results of syntheses	20a	For each synthesis, briefly summarise the characteristics and risk of bias among contributing studies.	6-48
	20b	Present results of all statistical syntheses conducted. If meta-analysis was done, present for each the summary estimate and its precision (e.g. confidence/credible interval) and measures of statistical heterogeneity. If comparing groups, describe the direction of the effect.	6-48
	20c	Present results of all investigations of possible causes of heterogeneity among study results.	6-48
	20d	Present results of all sensitivity analyses conducted to assess the robustness of the synthesized results.	6-48
Reporting biases	21	Present assessments of risk of bias due to missing results (arising from reporting biases) for each synthesis assessed.	6-48
Certainty of evidence	22	Present assessments of certainty (or confidence) in the body of evidence for each outcome assessed.	6-48
**DISCUSSION**			
Discussion	23a	Provide a general interpretation of the results in the context of other evidence.	48-66
	23b	Discuss any limitations of the evidence included in the review.	48-66
	23c	Discuss any limitations of the review processes used.	48-66

Table S1.PRISMA 2020 Checklist.			
**Section and Topic**	**Item #**	**Checklist item**	**Location where item is reported**
	23d	Discuss implications of the results for practice, policy, and future research.	48-66
**OTHER INFORMATION**			
Registration and protocol	24a	Provide registration information for the review, including register name and registration number, or state that the review was not registered.	4-6
	24b	Indicate where the review protocol can be accessed, or state that a protocol was not prepared.	4-6
	24c	Describe and explain any amendments to information provided at registration or in the protocol.	4-6
Support	25	Describe sources of financial or non-financial support for the review, and the role of the funders or sponsors in the review.	4-6
Competing interests	26	Declare any competing interests of review authors.	67
Availability of data, code and other materials	27	Report which of the following are publicly available and where they can be found: template data collection forms; data extracted from included studies; data used for all analyses; analytic code; any other materials used in the review.	67

*From:*Page MJ, McKenzie JE, Bossuyt PM, Boutron I, Hoffmann TC, Mulrow CD, et al. The PRISMA 2020 statement: an updated guideline for reporting systematic reviews. BMJ 2021;372:n71. doi: 10.1136/bmj.n71

For more information, visit: http://www.prisma-statement.org/

Table S2. Search Strategies.

Search database	Search terms	Number
**Pubmed**	(upadacitinib) AND ((autoimmune disease) OR (Disease, Autoimmune) OR (Addison Disease) OR (Anemia, Hemolytic, Autoimmune) OR (Anti-Glomerular Basement Membrane Disease) OR (Anti-Neutrophil Cytoplasmic Antibody-Associated Vasculitis) OR (Churg-Strauss Syndrome) OR (Antiphospholipid Syndrome) OR (Arthritis, Rheumatoid) OR (Rheumatoid Vasculitis) OR (Sjogren's Syndrome) OR (spondylarthritis) OR (Arthritis, Psoriatic) OR (Still's Disease, Adult-Onset) OR (Diabetes Mellitus, Type 1) OR (Graves Disease) OR (Hepatitis, Autoimmune) OR (Lupus Erythematosus, Systemic) OR (Lupus Nephritis) OR (Lupus Vasculitis, Central Nervous System) OR (Thyroiditis, Autoimmune) OR (Undifferentiated Connective Tissue Diseases) OR (Inflammatory Bowel Disease) OR (Colitis, Ulcerative) OR (Crohn Disease))	**481**
**Web of Science**	1. TS=(upadacitinib) and Preprint Citation Index (Exclude – Database)	**1131**
	2. ((((((((((((((((((((((((TS=(autoimmune disease)) OR TS=(Disease, Autoimmune)) OR TS=(Addison Disease)) OR TS=(Anemia, Hemolytic, Autoimmune)) OR TS=(Anti-Glomerular Basement Membrane Disease)) OR TS=(Anti-Neutrophil Cytoplasmic Antibody-Associated Vasculitis)) OR TS=(Churg-Strauss Syndrome)) OR TS=(Antiphospholipid Syndrome)) OR TS=(Arthritis, Rheumatoid)) OR TS=(Rheumatoid Vasculitis)) OR TS=(Sjogren's Syndrome)) OR TS=(spondylarthritis)) OR TS=(Arthritis, Psoriatic)) OR TS=(Still's Disease, Adult-Onset)) OR TS=(Diabetes Mellitus, Type 1)) OR TS=(Graves Disease)) OR TS=(Hepatitis, Autoimmune)) OR TS=(Lupus Erythematosus, Systemic)) OR TS=(Lupus Nephritis)) OR TS=(Lupus Vasculitis, Central Nervous System)) OR TS=(Thyroiditis, Autoimmune)) OR TS=(Undifferentiated Connective Tissue Diseases)) OR TS=(Inflammatory Bowel Disease)) OR TS=(Colitis, Ulcerative)) OR TS=(Crohn Disease) and Preprint Citation Index (Exclude – Database)	
	#2 AND #1	
**Embase**	1. 'upadacitinib'/exp OR upadacitinib	**2062**
	2. 'autoimmune disease'/exp OR 'autoimmune disease' OR (disease, AND autoimmune) OR 'addison disease' OR 'autoimmune hemolytic anemia' OR 'goodpasture syndrome' OR 'anca associated vasculitis' OR 'churg strauss syndrome' OR 'antiphospholipid syndrome' OR 'rheumatoid arthritis' OR 'rheumatoid vasculitis' OR 'sjoegren syndrome' OR 'spondylarthritis' OR 'psoriatic arthritis' OR 'adult onset still disease' OR 'insulin dependent diabetes mellitus' OR 'graves disease' OR 'autoimmune hepatitis' OR 'systemic lupus erythematosus' OR 'lupus erythematosus nephritis' OR 'central nervous system lupus' OR 'autoimmune thyroiditis' OR 'undifferentiated connective tissue disease' OR 'inflammatory bowel disease' OR 'ulcerative colitis' OR 'crohn disease'	
	#1 AND #2	

Table S3. Main characteristics of the included studies.

Disease	Study	Trial registration number	Country	Age range of the participants	Sample size		Intervention		Primary outcomes	Secondary outcomes	Duration
					Experimental group	Control group	Experimental group	Control group			
	Bergman et al. 2022[16]	NCT03086343	-	adults (18+)	303	309	UPA 15mg QD	abatacept (weight<60kg: 500mg, 60-100kg: 750mg, >100 kg: 1000 mg)	-	PtGA, HAQ-DI, FACIT-F, SF-36, pain VAS, etc.	24 weeks
	Burmester et al. 2018[18]	NCT02675426	in 35 countries	adults (18+)	221/219	221	UPA 15/30mg QD	placebo	ACR20, DAS28(CRP), AEs, etc.	ACR50/70, CDAI#, SDAI, HAQ-DI, etc.	12 weeks
	Burmester et al. 2024[19]	NCT02675426	-	adults (18+)	221/219	110/111	UPA 15/30mg QD	placebo→12week UPA 15/30mg QD	DAS28(CRP), AEs, etc.	CDAI#, etc.	260 weeks
	Charles-Schoeman et al. 2024[21]	NCT02706873 NCT02675426 NCT02706951 NCT02629159 NCT02706847 NCT03086343	-	adults (18+)	3209/1204	579/314	UPA 15/30mg QD	ADA 40mg EOW+MTX, MTX	AEs, etc.	laboratory parameters, etc.	240 weeks
	Conaghan et al. 2022[23]	NCT02629159	in 41 countries	-	651	651/327	UPA 15mg QD+MTX	placebo, ADA 40mg EOW	DAS28(CRP), AEs, etc.	CDAI#, SDAI, HAQ-DI, etc.	156 weeks
	Conaghan et al. 2023[24]	NCT02675426 NCT02629159 NCT02706951 NCT02706847	-	adults (18+)	209/649 72/144	195/671 73/143	UPA 15mg QD	placebo	ACR20, DAS28(CRP), etc.	ACR50/70, pain VAS, HAQ-DI, etc.	26 weeks
	Fleischmann et al. 2019[29]	NCT02629159	-	adults (18+)	651	651/327	UPA 15mg+MTX	placebo+MTX, ADA 40mg EOW+MTX	ACR20, DAS28(CRP), AEs, etc.	ACR50/70, CDAI#, SDAI, etc.	48 weeks
	Fleischmann et al. 2021[28]	NCT02629159	-	-	159	252	ADA→UPA 15mg	UPA 15mg→ADA	ACR20, DAS28(CRP), AEs, etc.	ACR50/70, CDAI#, etc.	24 weeks
	Fleischmann et al. 2022[26]	SELECT-COMPARE	-	-	651	651/327	UPA 15mg QD	placebo→26week UPA 15mg QD, ADA 40mg EOW→ 26week partially rescured to UPA 15mg	DAS28(CRP), AEs, etc.	CDAI#, pain VAS, mTSS, etc.	156 weeks
	Fleischmann et al. 2024[27]	NCT02629159	-	adults (18+)	651	651/327	UPA	placebo, ADA40mg EOW	AEs, etc.	clinical remission, radiographic progression, etc.	264 weeks
	Genovese et al. 2018[30]	NCT02706847	in 26 countries	adults (18+)	165/165	85/84	UPA 15/30mg QD	placebo→ UPA 15/30mg QD	ACR20, DAS28(CRP), AEs, etc.	ACR50/70, etc.	24 weeks

Disease	Study	Trial registration number	Country	Age range of the participants	Sample size		Intervention		Primary outcomes	Secondary outcomes	Duration
					Experimental group	Control group	Experimental group	Control group			
RA	Kameda et al. 2020[33]	NCT02720523	Japan	adults (18+)	49/49/50	49	UPA 7.5/15/30mg	placebo	ACR20, DAS28(CRP), AEs, etc.	ACR50/70, etc.	12 weeks
	Kameda et al. 2021[32]	NCT02720523	Japan	-	49/49/50	49	UPA 7.5/15/30mg	placebo→12week UPA 7.5/15/30mg	ACR20, AEs, etc.	ACR50/70, clinical remission, etc.	84 weeks
	Mysler et al. 2023[39]	NCT02629159	-	-	651	327	UPA 15mg QD	ADA 40mg EOW	DAS28(CRP), AEs, etc.	CDAI, pain VAS, etc.	48 weeks
	Pavelka et al. 2020[42]	NCT02629159	in 12 Central and Eastern European countries	adults (18+)	651	651/327	UPA 15mg QD	placebo, ADA 40mg EOW	ACR20, DAS28(CRP), AEs, etc.	ACR50/70, SF-36, pain VAS, etc.	48 weeks
	Peterfy et al. 2022[43]	NCT02706873 NCT02629159	-	-	317/314 651	314 651/327	MTX-naive: UPA 15/30mg QD MTX-IR： UPA 15mg QD	MTX-naive: MTX MTX-IR： placebo→ UPA 15mg QD/ ADA 40mg EOW	-	mTSS, etc.	48 weeks
	Smolen et al. 2019[49]	NCT02706951	in 24 countries	adults (18+)	217/215	216	UPA 15/30mg QD	MTX	ACR20, DAS28(CRP), AEs, etc.	ACR50/70, SF-36, pain VAS, etc.	14 weeks
	Strand, Tundia, Bergmanet et al. 2021[53]	NCT02629159	-	adults (18+)	651	651/327	UPA 15mg	placebo, ADA 40mg	-	SF-36, HAQ-DI, ptGA, pain VAS, morining stiffness, etc.	48 weeks
	Strand, Tundia, Wells et al. 2021[54]	NCT02706873 NCT02706951	-	adults (18+)	317/314 217/215	314/216	UPA 15/30mg	MTX	-	SF-36, HAQ-DI, ptGA, pain VAS, morining stiffness, etc.	14 weeks
	Strand, Pope et al. 2019[51]	NCT02675426	-	adults (18+)	221/219	221	UPA 15/30mg	placebo	-	PtGA, pain VAS, HAQ-DI, FACIT-F, morning stiffness, SF-36, etc.	12 weeks

Disease	Study	Trial registration number	Country	Age range of the participants	Sample size		Intervention		Primary outcomes	Secondary outcomes	Duration
					Experimental group	Control group	Experimental group	Control group			
	Strand, Schif et al. 2019[52]	NCT02706847	-	adults (18+)	164/165	169	UPA 15/30mg	placebo	-	PtGA, pain VAS, HAQ-DI, SF-36, morning stiffness, etc.	12 weeks
	Zeng et al. 2021[59]	NCT02955212	37 sites in China, Brazil, and South Korea	adults (18+)	169	169	UPA 15mg QD +csDMARDs	placebo+ csDMARDs	ACR20, DAS28(CRP), AEs, etc.	ACR50/70, SF-36, pain VAS, etc.	12 weeks
axSpA	Baral.iakos et al. 2023[15]	NCT02049138	-	adults (18+)	211	209	UPA 15mg QD	placebo→14week UPA 15mg QD	ASAS40, ASDAS ID/LDA, AEs, etc.	BASFI, etc.	52 weeks
	Van Der Heijde, Deodhar et al. 2022[57]	NCT03178487	-	adults (18+)	93	94	UPA 15mg QD	placebo→ UPA 15mg QD	ASAS40, ASDAS ID/LDA, AEs, etc.	back pain, etc.	104 weeks
	Van Der Heijde et al. 2019[58]	NCT03178487	in 20 countries	adults (18+)	93	94	UPA 15mg QD	placebo	ASAS40, ASDAS ID/LDA, AEs, etc.	back pain, etc.	14 weeks
	Van Der Heijde, Baral.iakos et al. 2022[56]	NCT04169373	in 22 countries	adults (18+)	211	209	UPA 15mg QD	placebo	ASAS20/40, BASDAI50, AEs, etc.	SPARCC MRI spine and sacroiliac joint scores, etc.	14 weeks
	Van den Bosch et al. 2024[55]	NCT04169373	-	adults (18+)	156	157	UPA 15mg QD	placebo	ASAS40, AEs, etc.	back pain, BASFI, hs-CRP, etc.	52 weeks
PsA	Burmester et al. 2022[20]	NCT03104400 NCT03104374	-	adults (18+)	907/921	635/429	UPA 15/30mg QD	placebo, ADA 40mg EOW	AEs, etc.	-	3 years(cut-off of June 29, 2020)
	McInnes et al. 2023[36]	NCT03104400	-	adults (18+)	214/210	211	UPA 15/30mg QD	ADA 40mg EOW	ACR20, AEs, etc.	ACR50/70, PASI75/90/100, MDA, mTSS, etc.	104 weeks
	Mease et al. 2021[38]	NCT03104374	-	-	211/219	106/106	UPA 15/30mg QD	placebo→ UPA 15/30mg QD	ACR20, AEs, etc.	ACR50/70, PASI75/90/100, HAQ-DI, etc.	56 weeks
	Nash et al. 2022[40]	NCT03104400 NCT03104374	-	-	189/197 353/341 451/444	188 342 447	UPA 15/30mg QD	placebo	ACR20, AEs, etc.	ACR50/70, PASI75/90/100, HAQ-DI, pain VAS, etc.	24 weeks
	Strand et al. (PsA) 2021[50]	NCT03104400	in 45 countries	adults (18+)	429/423	423/429	UPA 15/30mg QD	placebo/ ADA 40mg EOW	-	PtGA, HAQ-DI, FACIT-F, SF-36, MCID, etc.	56 weeks

Disease	Study	Trial registration number	Country	Age range of the participants	Sample size		Intervention		Primary outcomes	Secondary outcomes	Duration
					Experimental group	Control group	Experimental group	Control group			
CD	Colombel et al. 2024[22]	NCT03345849<br>NCT03345836<br>NCT03345823	-	adults (18-75)	674	347	UPA 45mg QD	placebo	-	SF, APS, CDAI*, etc.	12 weeks
	Loftus et al. (CD) 2023[35]	NCT03345849<br>NCT03345836<br>NCT03345823	in 43 countries	adults (18-75)	350<br>324<br>169/168	176<br>171<br>165	U-EXCEL introduction: UPA 45mg<br>U-EXCEED introduction: UPA 45mg<br>U-ENDURE maintenance： UPA 15/30mg	placebo	clinical remission, endoscopic response, AEs, etc.	endoscopic remission, IBDQ, FACIT-F, etc.	52 weeks
	Peyrin-Biroulet et al. 2021[44]	NCT02365649	-	adults (18-75)	39/37/36/36/35	37	UPA 3/6/12/24mg BID+24mg QD→ 16week rerandomized to UPA 3/6/12mg BID+24mg QD	placebo→16week rerandomized to UPA 3/6/12mg BID+24mg QD	-	IBDQ,<br>EQ-5D VAS,<br>WPAI, etc.	52 weeks
	Peyrin-Biroulet et al. 2024[45]	NCT03345849<br>NCT03345836<br>NCT03345823	-	adults (18-75)	189/485	99/248	UPA 15/30/45mg with or without prior biologic failure	placebo	clinical remission, endoscopic response, AEs, etc.	-	introduction/ maintenance period： 12/52weeks
	Sandborn et al. (CD) 2020[47]	NCT02365649	in 19 countries	adults (18-75)	39/37/36/36/35	37	UPA 3/6/12/24mg BID+24mg QD	placebo	clinical remission, endoscopic response, AEs, etc.	hs-CRP, IBDQ, etc.	52 weeks
UC	Danese et al. 2023[25]	NCT02819635<br>NCT03653026	-	-	660	328	UPA 45mg QD→ responders assigned to PBO or UPA 15/30mg	placebo	UC symptoms, etc.	FACIT-F, etc.	52 weeks
	Ghosh et al. 2021[31]	U-ACHIEVE	-	adults (18-75)	47/49/52/56	46	UPA 7.5/15/30/45mg QD	placebo	UC symptoms, etc.	QOL,<br>SF-36, hs-CRP,<br>IBDQ, etc.	8 weeks
	Loftus et al. (UC) 2023[34]	NCT02819635<br>NCT03653026	-	-	660	328	UPA 45mg QD	placebo	UC symptoms, , clinical remission, etc.	clinical response, hs-CRP, FCP, QOL, etc	8 weeks
	Panés et al. 2023[41]	NCT02819635<br>NCT03653026	-	patients (16-75)	660	328	UPA 45mg QD	placebo	-	UC-SQ, IBDQ, WPAI, EQ-5D, etc.	introduction/ maintenance period： 8/52weeks
	Sandborn et al. (UC) 2020[48]	NCT02819635	in 28 countries	adults (18-75)	47/49/52/56	46	UPA 7.5/15/30/45mg QD	placebo	AEs, etc.	clinical remission, endoscopic remission, etc.	8 weeks

Disease	Study	Trial registration number	Country	Age range of the participants	Sample size		Intervention		Primary outcomes	Secondary outcomes	Duration
					Experimental group	Control group	Experimental group	Control group			
RA, AS, PsA, AD	Burmester et al. 2023[17]	NCT02675426<br>NCT02706951<br>NCT02706847<br>NCT02629159<br>NCT02706873<br>NCT03086343<br>NCT03104374<br>NCT03104400<br>NCT03178487<br>NCT03569293<br>NCT03568318<br>NCT03607422	-	adults (18+)	3209<br>907<br>182<br>1340/1353	579/314<br>429<br>-<br>-	RA:UPA 15mg QD<br>PsA:UPA 15mg QD<br>AS:UPA 15mg QD<br>AD:UPA 15/30mg QD	RA:ADA 40mg EOW/ MTX<br>PsA:ADA 40mg EOW<br>AS:-<br>AD:-	AEs	-	-<br>cutoff 30 June 2021
RA, AS, PsA, nr-axSpA	Rubbert-Roth et al. 2024[46]	NCT02706873<br>NCT02675426<br>NCT02629159<br>NCT02706951<br>NCT02706847<br>NCT03086343<br>NCT03104400<br>NCT03104374<br>NCT03178487<br>NCT04169373	-	adults (18+)	4998/2125	1008/314	UPA 15/30mg	ADA, MTX	rates of malignancy	-	-<br>cutoff 15 Aug 2022
AS, PsA	McInnes et al. 2022[37]	NCT03104400<br>NCT03104374<br>NCT03178487	-	adults (18+)	429<br>211<br>93	211/429<br>106<br>94	UPA 15mg QD	SELECT-PSA1：placebo→ UPA 15mg QD/ ADA 40mg EOW<br>SELECT-PSA2: placebo→ UPA 15mg QD<br>SELECT-AXIS1: placebo→ UPA 15mg QD	-	pain reduction	56 weeks

Abbreviations: ACR20/50/70: At least 20%/50%/70% improvement in American College of Rheumatology response criteria; ADA: Adalimumab; AD: Atopic Dermatitis; AEs: Adverse Events; APS: Abdominal Pain Score; ASAS20/40: At least 20%/40% improvement in Assessment of SpondyloArthritis International Society; ASDAS: Ankylosing Spondylitis Disease Activity; axSpA: Axial Spondyloarthritis; BASDAI50: At least 50% improvement in Bath Ankylosing Spondylitis Disease Activity Index; BASFI: Bath Ankylosing Spondylitis Functional Index; BID: Twice Daily; CD: Crohn’s Disease; CDAI:* Crohn’s Disease Activity Index; CDAI#: Clinical Disease Activity Index; DAS28(CRP): 28-Joint Disease Activity Score using C-reactive Protein; EQ-5D: European Quality of Life-5 Dimensions; EOW: Every Other Week; FACIT-F: Functional Assessment of Chronic Illness Therapy–Fatigue; FCP: Fecal Calprotectin; HAQ-DI: Health Assessment Questionnaire-Disability Index; hs-CRP: High-sensitivity C-reactive Protein; IBQD: Inflammatory Bowel Disease Questionnaire; ID: Inactive Disease; LDA: Low Disease Activity; MDA: Minimal Disease Activity; MCID: Minimal Clinically Important Differences; mTSS: Modified Total Sharp/van der Heijde Score; MTX: Methotrexate; nr-axSpA: Non-radiographic Axial Spondyloarthritis; PASI75/90/100: At least 75%, 90%, or 100% improvement in the Psoriasis Area Severity Index; PsA: Psoriatic Arthritis; PtGA: Patient’s Global Assessment of Disease Activity; QOL: Quality of Life; QD: Once Daily; RA: Rheumatoid Arthritis; SDAI: Simplified Disease Activity Index; SF: Stool Frequency; SF-36: 36-Item Short Form Health Survey; SPARCC: Spondyloarthritis Research Consortium of Canada; UC: Ulcerative Colitis; UC-SQ: Ulcerative Colitis Symptoms Questionnaire; UPA: Upadacitinib; VAS: Visual Analog Scale; WPAI: Work Productivity and Activity Impairment Questionnaire.

Table S4. Efficacy Outcomes Summary: Upadacitinib vs Non-Upadacitinib in RA, axSpA, PsA, CD, and UC.

Disease	Outcome	Number of articles	Upadacitinib	Control group	Risk Ratio(95%CI)	Mean difference (95%CI)	P-value	Heterogeneity (I²)	Effects model
RA	ACR20	6	15mg QD	placebo	1.93 [1.80,2.06]	-	<0.001	0%	Fixed
		3	30mg QD	placebo	1.90 [1.64,2.20]	-	<0.001	0%	Fixed
		5	15mg QD	ADA 40mg EOW	1.24 [1.16,1.32]	-	<0.001	0%	Fixed
		1	15mg QD	MTX	1.64 [1.37,1.98]	-	-	-	-
		1	30mg QD	MTX	1.73 [1.44,2.07]	-	-	-	-
	ACR50	6	15mg QD	placebo	2.95 [2.60,3.36]	-	<0.001	0%	Fixed
		3	30mg QD	placebo	3.01 [2.33,3.90]	-	<0.001	0%	Fixed
		5	15mg QD	ADA 40mg EOW	1.33 [1.22,1.44]	-	<0.001	0%	Fixed
		1	15mg QD	MTX	2.74 [1.93,3.90]	-	-	-	-
		1	30mg QD	MTX	3.41 [2.43,4.79]	-	-	-	-
	ACR70	6	15mg QD	placebo	4.38 [3.53,5.43]	-	<0.001	36%	Fixed
		3	30mg QD	placebo	4.50 [2.97,6.80]	-	<0.001	0%	Fixed
		5	15mg QD	ADA 40mg EOW	1.51 [1.35,1.69]	-	<0.001	0%	Fixed
		1	15mg QD	MTX	8.13 [3.56,18.58]	-	-	-	-
		1	30mg QD	MTX	11.89 [5.28,26.77]	-	-	-	-
	DAS28(CRP) LDA	6	15mg QD	placebo	2.92 [2.41,3.53]	-	<0.001	62%	Random
		3	30mg QD	placebo	2.85 [2.20,3.69]	-	<0.001	0%	Fixed
		6	15mg QD	ADA 40mg EOW	1.36 [1.26,1.47]	-	<0.001	1%	Fixed
		1	15mg QD	MTX	2.38 [1.74,3.25]	-	-	-	-
		1	30mg QD	MTX	2.79 [2.06,3.78]	-	-	-	-
	DAS28(CRP) CR	5	15mg QD	placebo	4.12 [3.46,4.92]	-	<0.001	47%	Fixed
		2	30mg QD	placebo	4.24 [1.53,11.70]	-	0.005	66%	Random
		6	15mg QD	ADA 40mg EOW	1.64 [1.33,2.03]	-	<0.001	76%	Random
		1	15mg QD	MTX	3.57 [2.16,5.91]	-	-	-	-
		1	30mg QD	MTX	5.20 [3.21,8.44]	-	-	-	-
	CDAI# LDA	5	15mg QD	placebo	2.55 [2.15,3.02]	-	<0.001	11%	Fixed
		3	30mg QD	placebo	2.67 [1.74,4.08]	-	<0.001	60%	Random
		6	15mg QD	ADA 40mg EOW	1.31 [1.21,1.41]	-	<0.001	0%	Fixed
		1	15mg QD	MTX	1.40 [1.04,1.88]	-	-	-	-
		1	30mg QD	MTX	1.88 [1.43,2.46]	-	-	-	-
	CDAI# clinical remission	4	15mg QD	placebo	4.99 [3.05,8.17]	-	<0.001	0%	Fixed
		2	30mg QD	placebo	4.39 [2.07,9.30]	-	<0.001	0%	Fixed
		6	15mg QD	ADA 40mg EOW	1.50 [1.32,1.71]	-	<0.001	19%	Fixed
		1	15mg QD	MTX	13.94 [3.36,57.78]	-	-	-	-
		1	30mg QD	MTX	20.60 [5.04,84.08]	-	-	-	-
	SDAI LDA	4	15mg QD	placebo	2.47 [2.06,2.96]	-	<0.001	0%	Fixed
		2	30mg QD	placebo	2.81 [1.94,4.06]	-	<0.001	51%	Random
		1	15mg QD	ADA 40mg EOW	1.34 [1.11,1.63]	-	-	-	-
		1	15mg QD	MTX	1.53 [1.14,2.05]	-	-	-	-
		1	30mg QD	MTX	1.95 [1.48,2.57]	-	-	-	-

Disease	Outcome	Number of articles	Upadacitinib	Control group	Risk Ratio(95%CI)	Mean difference (95%CI)	P-value	Heterogeneity (I^2)	Effects model
RA	SDAI clinical remission	3	15mg QD	placebo	4.77 [2.91,7.80]	-	<0.001	0%	Fixed
		2	30mg QD	placebo	4.01 [1.97,8.16]	-	0.25	57%	Random
		2	15mg QD	ADA 40mg EOW	1.67 [1.34,2.09]]	-	<0.001	21%	Fixed
		1	15mg QD	MTX	14.93 [3.61,1.70]	-	-	-	-
		1	30mg QD	MTX	19.59 [4.79,0.11]	-	-	-	-
	change in PAIN (VAS 0-100)	1	15mg QD	abatacept	-	-3.80 [-8.47,0.87]	-	-	-
		1	15mg QD	ADA 40mg EOW	-	1.14 [-0.73,3.00]	-	-	-
		2	15mg QD	placebo	-	-9.61 [-26.86,7.64]	0.28	99%	Random
	change in HAQ-DI	1	15mg QD	abatacept	-	-0.13 [-0.25,-0.01]	-	-	-
		7	15mg QD	placebo	-	-0.31 [-0.34,-0.27]	<0.001	9%	Fixed
		4	30mg QD	placebo	-	-0.28 [-0.35,-0.22]	<0.001	0%	Fixed
		5	15mg QD	ADA 40mg EOW	-	-0.11 [-0.16,-0.06]	<0.001	0%	Fixed
		2	15mg QD	MTX	-	-0.33 [-0.41,-0.25]	<0.001	0%	Fixed
		2	30mg QD	MTX	-	-0.41 [-0.49,-0.33]	<0.001	0%	Fixed
	change in morining stiffness duration	1	15mg QD	abatacept	-	-4.30 [-56.45,47.85]	-	-	-
		6	15mg QD	placebo	-	-48.21 [-58.63,-37.79]	<0.001	3%	Fixed
		4	30mg QD	placebo	-	-56.14 [-71.94,-40.34]	<0.001	0%	Fixed
		2	15mg QD	ADA 40mg EOW	-	-8.15 [-20.33,4.03]	0.19	0%	Fixed
		2	15mg QD	MTX	-	-41.56 [-60.66,-22.47]	<0.001	0%	Fixed
		2	30mg QD	MTX	-	-49.30 [-68.35,-30.26]	<0.001	0%	Fixed
	change in morining stiffness severity	1	15mg QD	abatacept	-	-0.40 [-0.82,0.02]	-	-	-
		4	15mg QD	placebo	-	-1.51 [-1.76,-1.26]	<0.001	0%	Fixed
		2	30mg QD	placebo	-	-1.78 [-2.16,-1.41]	<0.001	0%	Fixed
		2	15mg QD	ADA 40mg EOW	-	-0.43 [-0.72,-0.15]	0.003	0%	Fixed
		1	15mg QD	MTX	-	-0.70 [-1.10,-0.30]	-	-	-
		1	30mg QD	MTX	-	-1.04 [-1.44,-0.64]	-	-	-
	SF-36 PCS	1	15mg QD	abatacept	-	1.60 [0.01,3.19]	-	-	-
		6	15mg QD	placebo		4.37 [3.59,5.14]	<0.001	0%	Fixed
		3	30mg QD	placebo		4.82 [3.62,6.02]	<0.001	0%	Fixed
		3	15mg QD	ADA 40mg EOW		1.71 [0.82,2.60]	<0.001	0%	Fixed
		2	15mg QD	MTX		3.97 [3.07,4.88]	<0.001	0%	Fixed
		2	30mg QD	MTX		5.88 [4.97,6.79]	<0.001	0%	Fixed
	SF-36 MCS	1	15mg QD	abatacept	-	0.00 [-1.73,1.73]	-	-	-
		3	15mg QD	placebo	-	2.30 [1.39,3.20]	<0.001	0%	Fixed
		2	30mg QD	placebo	-	0.74 [-0.58,2.06]	0.27	0%	Fixed
		1	15mg QD	ADA 40mg EOW	-	0.73 [-0.36,1.82]	-	-	-
		1	15mg QD	MTX	-	2.67 [0.93,4.41]	-	-	-
		1	30mg QD	MTX	-	2.80 [1.06,4.54]	-	-	-

Disease	Outcome	Number of articles	Upadacitinib	Control group	Risk Ratio(95%CI)	Mean difference (95%CI)	P-value	Heterogeneity (I^2)	Effects model
	△mTSS ≤0	5	15mg QD	placebo	1.14 [1.11, 1.18]	-	<0.001	28%	Fixed
		5	15mg QD	ADA 40mg EOW	0.99 [0.96, 1.03]	-	0.72	0%	Fixed
		1	15mg QD	MTX	1.18 [1.12, 1.25]	-	<0.001	0%	Fixed
		1	30mg QD	MTX	1.20 [1.13, 1.27]	-	<0.001	9%	Fixed
axSpA	ASAS20	5	15mg QD	placebo	1.28 [1.01,1.61]		0.04	88%	Random
	ASAS40	5	15mg QD	placebo	1.47 [1.05,2.04]	-	0.02	90%	Random
	ASDAS ID	5	15mg QD	placebo	2.24 [1.05,4.78]	-	0.04	88%	Random
	ASDAS LDA	5	15mg QD	placebo	1.40 [1.02,1.91]	-	0.03	88%	Random
	BASDAI50	5	15mg QD	placebo	1.47 [1.10,1.97]	-	0.01	85%	Random
	change in total back pain	3	15mg QD	placebo	-	-0.80 [-1.56,-0.05]	0.04	81%	Random
	change in nocturnal back pain	3	15mg QD	placebo	-	-0.72 [-1.55,0.11]	0.09	83%	Random
	change in BASFI	4	15mg QD	placebo	-	-0.70 [-1.18,-0.22]	0.004	67%	Random
	△SPARCC MRI spine scores	2	15mg QD	placebo	-	-0.73 [-1.19, -0.28]	0.002	69%	Random
	△SPARCC MRI sacroiliac joint scores	2	15mg QD	placebo	-	-0.80 [-1.29, -0.30]	0.002	73%	Random
PsA	ACR20	2	15mg QD	placebo	2.46 [1.86,3.26]	-	<0.001	87%	Random
		2	30mg QD	placebo	2.68 [2.02,3.54]	-	<0.001	87%	Random
		1	15mg QD	ADA 40mg EOW	1.09 [0.99,1.20]	-	-	-	-
		1	30mg QD	ADA 40mg EOW	1.10 [1.00,1.21]	-	-	-	-
	ACR50	2	15mg QD	placebo	3.98 [2.78,5.70]	-	<0.001	71%	Random
		2	30mg QD	placebo	4.70 [3.48,6.36]	-	<0.001	60%	Random
		1	15mg QD	ADA 40mg EOW	1.14 [1.00,1.30]	-	-	-	-
		1	30mg QD	ADA 40mg EOW	1.26 [1.11,1.43]	-	-	-	-
	ACR70	2	15mg QD	placebo	9.41 [4.21,21.00]	-	<0.001	57%	Random
		2	30mg QD	placebo	13.66 [8.91,20.94]	-	<0.001	43%	Fixed
		1	15mg QD	ADA 40mg EOW	1.29 [1.07,1.56]	-	-	-	-
		1	30mg QD	ADA 40mg EOW	1.48 [1.23,1.78]	-	-	-	-
	PASI75	2	15mg QD	placebo	2.93 [2.29,3.75]	-	<0.001	51%	Random
		2	30mg QD	placebo	3.19 [2.17,4.67]	-	<0.001	79%	Random
		1	15mg QD	ADA 40mg EOW	0.99 [0.84,1.16]	-	-	-	-
		1	30mg QD	ADA 40mg EOW	1.06 [0.91,1.24]	-	-	-	-
	PASI90	2	15mg QD	placebo	3.42 [2.71,4.33]	-	<0.001	0%	Fixed
		2	30mg QD	placebo	4.32 [3.44,5.44]	-	<0.001	46%	Fixed
		1	15mg QD	ADA 40mg EOW	0.96 [0.78,1.17]	-	-	-	-
		1	30mg QD	ADA 40mg EOW	1.09 [0.91,1.32]	-	-	-	-

Disease	Outcome	Number of articles	Upadacitinib	Control group	Risk Ratio(95%CI)	Mean difference (95%CI)	P-value	Heterogeneity (I²)	Effects model
PsA	PASI100	2	15mg QD	placebo	4.58 [3.38,6.20]	-	<0.001	32%	Fixed
		2	30mg QD	placebo	4.09 [3.01,5.56]	-	<0.001	0%	Fixed
		1	15mg QD	ADA 40mg EOW	1.00 [0.77,1.30]	-	-	-	-
		1	30mg QD	ADA 40mg EOW	1.20 [0.94,1.54]	-	-	-	-
	LEI=0	2	15mg QD	placebo	1.58 [1.08,2.33]	-	0.02	88%	Random
		2	30mg QD	placebo	1.77 [1.36,2.30]	-	<0.001	73%	Random
		1	15mg QD	ADA 40mg EOW	1.09 [0.92,1.28]	-	-	-	-
		1	30mg QD	ADA 40mg EOW	1.06 [0.90,1.26]	-	-	-	-
	LDI=0	2	15mg QD	placebo	1.62 [0.84,3.11]	-	0.15	96%	Random
		2	30mg QD	placebo	1.76 [0.83,3.72]	-	0.14	98%	Random
		1	15mg QD	ADA 40mg EOW	0.96 [0.83,1.12]	-	-	-	-
		1	30mg QD	ADA 40mg EOW	0.96 [0.56,1.66]	-	-	-	-
	MDA	2	15mg QD	placebo	4.97 [2.71,9.09]	-	<0.001	85%	Random
		2	30mg QD	placebo	6.04 [3.22,11.31]	-	<0.001	86%	Random
		1	15mg QD	ADA 40mg EOW	1.11 [0.94,1.31]	-	-	-	-
		1	30mg QD	ADA 40mg EOW	1.21 [1.04,1.42]	-	-	-	-
	change in BASDAI	3	15mg QD	placebo	-	-0.53 [-1.51,0.45]	0.29	82%	Random
		3	30mg QD	placebo	-	-0.57 [-1.64,0.49]	0.29	83%	Random
		2	15mg QD	ADA 40mg EOW	-	-0.51 [-0.97,-0.05]	0.03	0	Fixed
		2	30mg QD	ADA 40mg EOW	-	-0.74 [-1.20,-0.27]	0.002	0	Fixed
	change in PAIN (NRS 0-10)	4	15mg QD	placebo	-	-1.19 [-1.35,-1.03]	<0.001	91%	Random
		4	30mg QD	placebo	-	-1.59 [-1.74,-1.44]	<0.001	91%	Random
		2	15mg QD	ADA 40mg EOW	-	-0.41 [-0.82,0.01]	0.06	0	Fixed
		2	30mg QD	ADA 40mg EOW	-	-0.30 [-0.69,0.08]	0.13	0	Fixed
	change in HAQ-DI	4	15mg QD	placebo	-	-0.21 [-0.30,-0.12]	<0.001	85%	Random
		4	30mg QD	placebo	-	-0.26 [-0.34,-0.17]	<0.001	82%	Random
		2	15mg QD	ADA 40mg EOW	-	-0.11 [-0.17,-0.05]	<0.001	0	Fixed
		2	30mg QD	ADA 40mg EOW	-	-0.12 [-0.18,-0.06]	<0.001	0	Fixed
CD	SF/APS clinical remission	3	45mg QD	placebo	2.47 [2.12,2.88]	-	<0.001	9%	Fixed
		2	30mg QD	placebo	2.97 [1.72,5.12]	-	<0.001	72%	Random
		2	15mg QD	placebo	2.27 [1.33,3.89]	-	0.003	68%	Random
	CDAI* clinical remission	3	45mg QD	placebo	1.71 [1.51,1.95]	-	<0.001	23%	Fixed
		2	30mg QD	placebo	2.97 [1.72,5.12]	-	<0.001	14%	Fixed
		2	15mg QD	placebo	2.78 [2.09,3.68]	-	<0.001	8%	Fixed
	endoscopic response	3	45mg QD	placebo	4.79 [3.18,7.20]	-	<0.001	60%	Random
		2	30mg QD	placebo	4.14 [1.97,8.70]	-	<0.001	66%	Random
		2	15mg QD	placebo	3.39 [2.22,5.16]	-	<0.001	0%	Fixed

Disease	Outcome	Number of articles	Upadacitinib	Control group	Risk Ratio(95%CI)	Mean difference (95%CI)	P-value	Heterogeneity (I^2)	Effects model
UC	clinical remission	2	45mg QD	placebo	3.77 [2.91,4.88]	-	<0.001	0%	Fixed
	SFS≤1	3	45mg QD	placebo	2.41 [1.97,2.95]	-	<0.001	0%	Fixed
	RBS of 0	3	45mg QD	placebo	2.47 [2.07,2.96]	-	<0.001	0%	Fixed
	APS of 0	3	45mg QD	placebo	1.94 [1.68,2.24]	-	<0.001	30%	Fixed
	Bowel urgency absence	3	45mg QD	placebo	2.33 [1.79,3.02]	-	<0.001	58%	Random

Abbreviations: ACR20/50/70: At least 20%/50%/70% improvement in American College of Rheumatology Response Criteria; ADA: Adalimumab; APS: Abdominal Pain Score; ASAS20/40: At least 20%/40% improvement in Assessment of SpondyloArthritis International Society; ASDAS: Ankylosing Spondylitis Disease Activity; axSpA: Axial Spondyloarthritis; BASDAI50: At least 50% improvement in Bath Ankylosing Spondylitis Disease Activity Index; BASFI: Bath Ankylosing Spondylitis Functional Index; CD: Crohn's Disease; CDAI*: Crohn's Disease Activity Index; CDAI#: Clinical Disease Activity Index; CR: Clinical Remission; DAS28(CRP): 28-Joint Disease Activity Score using C-reactive Protein; EOW: Every Other Week; HAQ-DI: Health Assessment Questionnaire-Disability Index; ID: Inactive Disease; LDA: Low Disease Activity; LDI: Leeds Dactylitis Index; LEI: Leeds Enthesitis Index; MCS: Mental Component Summary (SF-36); MDA: Minimal Disease Activity; mTSS: Modified Total Sharp/van der Heijde Score; MTX: Methotrexate; PASI75/90/100: At least 75%, 90%, or 100% improvement in the Psoriasis Area Severity Index ; PCS: Physical Component Summary (SF-36); PsA:Psoriatic Arthritis; QD: Once Daily; RA: Rheumatoid Arthritis; RBS: Rectal Bleeding Score; SDAI: Simplified Disease Activity Index; SF: Stool Frequency; SF-36: 36-Item Short Form Health Survey; SFS: Stool Frequency Score; SPARCC: Spondyloarthritis Research Consortium of Canada; UC: Ulcerative Colitis.
