# Supplementary material for: Efficacy and safety of upadacitinib for patients with immune-mediated inflammatory diseases: a systematic review and meta-analysis
Source: Front Immunol. 2025 Jul 1;16:1586792. doi: 10.3389/fimmu.2025.1586792 (PMC12259590; doi:10.3389/fimmu.2025.1586792)
Supplement: Supplementary file 2 [file DataSheet2.pdf]

Additional file 2

Fig S1. Risk of bias summary: review authors' judgements about each risk of bias item for each included study.

Fig S2. Key efficacy outcomes of upadacitinib versus placebo or ADA for RA.

Fig S3. ACR20 of upadacitinib 30 mg QD versus placebo for PsA.

Fig S4. Specific AEs of upadacitinib with recommended doses versus non-upadacitinib therapies.

Fig S5. Overall AEs assessed by different doses of upadacitinib.

	Random sequence generation (selection bias)	Allocation concealment (selection bias)	Blinding of participants and personnel (performance bias)	Blinding of outcome assessment (detection bias)	Incomplete outcome data (attrition bias)	Selective reporting (reporting bias)	Other bias
Baraliakos et al 2023	+	+	−	+	+	?	?
Bergman et al 2022	?	+	−	−	+	−	−
Burmester et al 2018	?	+	+	+	+	+	+
Burmester et al 2022	?	+	−	−	+	+	+
Burmester et al 2023	?	+	−	−	+	+	?
Burmester et al 2024	?	+	+	+	+	+	−
Charles-Schoeman et al 2024	+	+	+	−	+	?	?
Colombel et al 2024	+	+	+	+	+	+	+
Conaghan et al 2022	?	+	−	−	+	+	?
Conaghan et al 2023	?	+	+	+	+	+	−
Danese et al 2023	?	+	+	+	+	+	?
Fleischmann et al 2019	+	+	+	+	+	+	+
Fleischmann et al 2021	?	+	+	+	+	+	+
Fleischmann et al 2022	?	+	−	−	+	+	?
Fleischmann et al 2024	?	+	−	−	+	+	?
Genovese et al 2018	+	+	+	+	+	+	+
Ghosh et al 2021	?	+	−	−	+	+	?
Kameda et al 2020	+	+	+	+	+	+	+
Kameda et al 2021	+	+	+	+	+	+	+
Loftus et al (CD) 2023	+	+	+	+	+	+	+
Loftus et al (UC) 2023	+	+	−	−	+	+	−
McInnes et al 2022	?	+	−	+	+	+	?
McInnes et al 2023	?	+	+	+	+	+	−
Mease et al 2021	?	+	−	−	+	+	?
Mysler et al 2023	?	+	+	+	+	+	?
Nash et al 2022	?	+	−	−	+	−	−
Panés et al 2023	?	+	−	−	+	+	?
Pavelka et al 2020	?	+	−	−	+	+	?
Peterfy et al 2022	?	+	+	+	+	+	?
Peyrin-Biroulet et al 2021	+	+	+	+	+	+	+
Peyrin-Briouet et al 2024	+	+	+	+	+	+	+
Rubbert-Roth et al 2024	?	+	−	−	+	+	−
Sandborn et al (CD) 2020	+	+	+	+	+	+	+
Sandborn et al (UC) 2020	+	+	+	+	+	+	+
Smolen et al 2019	+	+	+	+	+	+	+
Strand, Pope et al 2019	?	+	+	+	+	+	?
Strand, Schif et al 2019	?	+	+	+	+	+	?
Strand, Tundia, Bergmanet et al 2021	?	+	+	+	+	+	+
Strand, Tundia, Wells et al 2021	?	+	+	+	+	+	?
Strand et al (PsA) 2021	?	+	+	+	+	+	?
van den Bosch et al 2024	?	+	−	−	+	+	?
van der Heijde, Baraliakos et al 2022	+	+	+	+	+	+	+
Van Der Heijde, Deodhar et al 2022	?	+	−	−	+	+	?
van der Heijde et al 2019	+	+	+	+	+	+	+
Zeng et al 2021	+	+	+	+	+	+	+

Fig S1. Risk of bias summary: review authors' judgements about each risk of bias item for each included study.

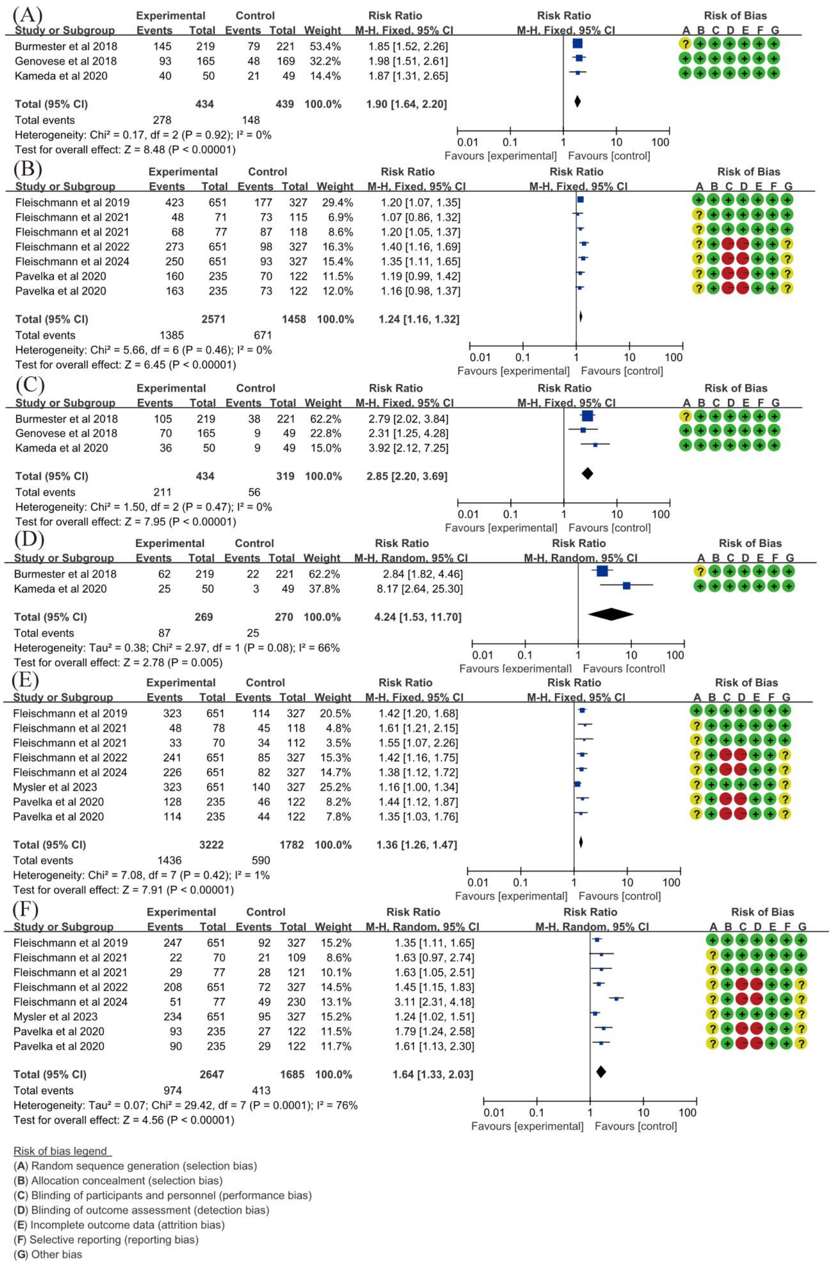

Fig S2. Key efficacy outcomes of upadacitinib versus placebo or ADA for RA.

(A) ACR20: upadacitinib 30 mg QD vs placebo; (B) ACR20: upadacitinib 15 mg QD vs ADA 40 mg EOW; (C) DAS28(CRP) LDA: upadacitinib 30 mg QD vs placebo; (D) DAS28(CRP) LDA: upadacitinib 15 mg QD vs ADA 40 mg EOW; (E) DAS28(CRP) CR: upadacitinib 30 mg QD vs placebo; (F) DAS28(CRP) CR: upadacitinib 15 mg QD vs ADA 40 mg EOW.

Experimental: upadacitinib 15/30 mg QD; Control: placebo or ADA 40 mg EOW.

The appearance of the same study is due to subgroup analyses or pooled analyses of different RCTs performed on the same experimental dose and control group with no duplication analyses.

Abbreviations: ACR20, at least 20% improvement in American College of Rheumatology Response Criteria; ADA: adalimumab; CR, clinical remission; DAS28(CRP), 28-joint Disease Activity Score using C-reactive Protein; EOW, every other week; LDA, low disease activity; QD, once daily; RA, rheumatoid arthritis.

Study or Subgroup	Experimental Events	Experimental Total	Control Events	Control Total	Weight	Risk Ratio M-H, Random, 95% CI
Mease et al 2021	134	218	43	312	22.1%	4.46 [3.31, 6.00]
Nash et al 2022	254	341	120	342	27.0%	2.12 [1.81, 2.48]
Nash et al 2022	332	444	157	447	27.5%	2.13 [1.86, 2.44]
Nash et al 2022	139	197	47	188	23.4%	2.82 [2.17, 3.67]
**Total (95% CI)**		**1200**		**1289**	**100.0%**	**2.68 [2.02, 3.54]**
Total events	859		367			

Heterogeneity: Tau² = 0.07; Chi² = 23.98, df = 3 (P < 0.0001); I² = 87%

Test for overall effect: Z = 6.91 (P < 0.00001)

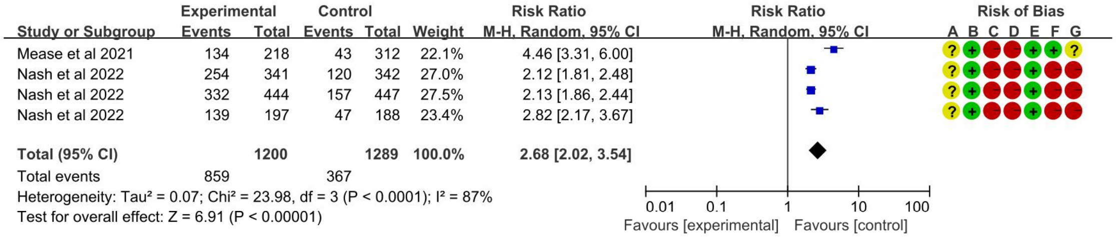

Risk of bias legend

(**A**) Random sequence generation (selection bias)
(**B**) Allocation concealment (selection bias)
(**C**) Blinding of participants and personnel (performance bias)
(**D**) Blinding of outcome assessment (detection bias)
(**E**) Incomplete outcome data (attrition bias)
(**F**) Selective reporting (reporting bias)
(**G**) Other bias

Fig S3. ACR20 of upadacitinib 30 mg QD versus placebo for PsA.

Experimental: upadacitinib 30 mg QD; Control: placebo.

The appearance of the same study is due to subgroup analyses or pooled analyses of different RCTs performed on the same experimental dose and control group with no duplication analyses.

Abbreviations: ACR20, at least 20% improvement in American College of Rheumatology Response Criteria; PsA, Psoriatic Arthritis; QD, once daily.

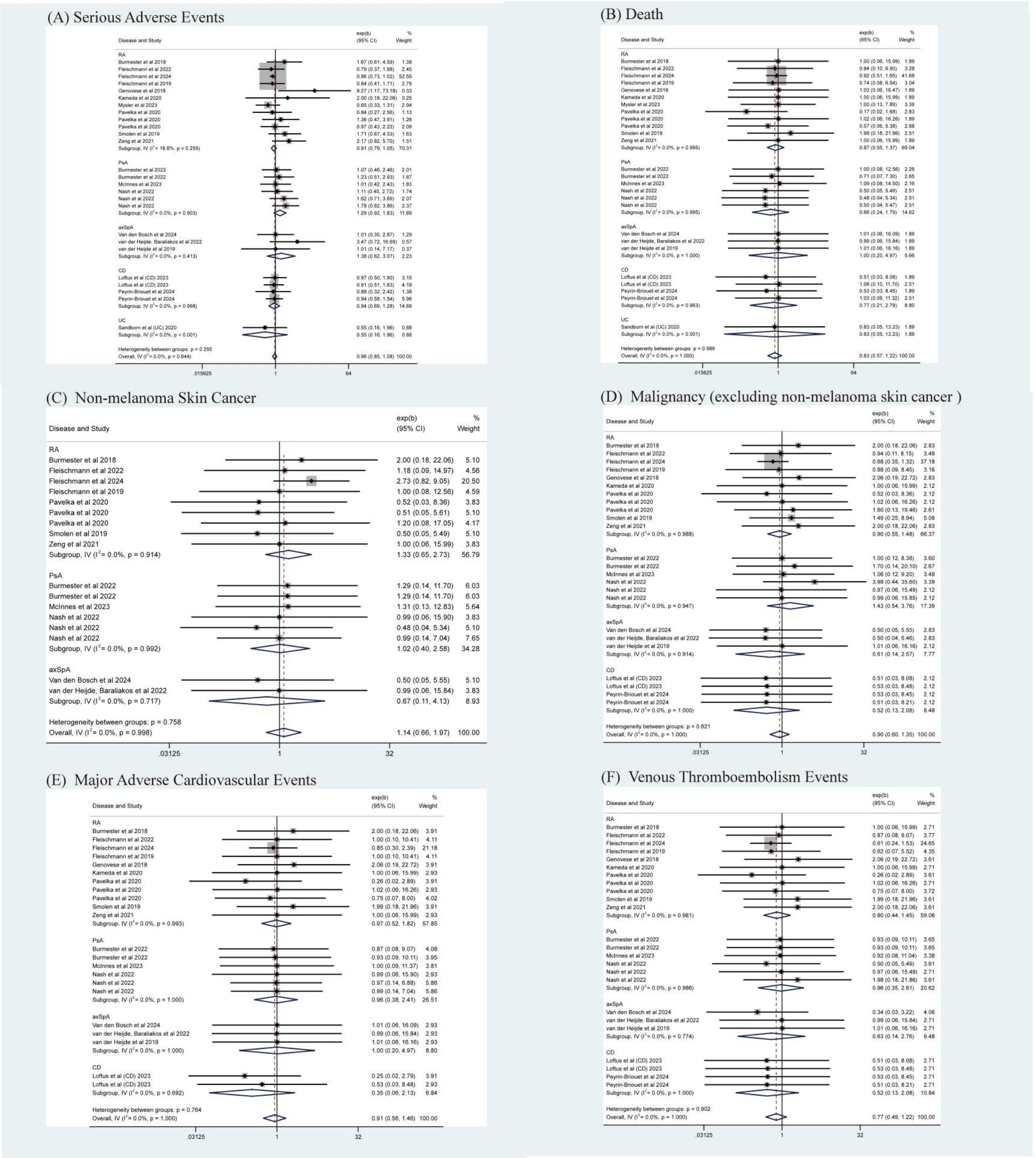

Fig S4. Specific AEs of upadacitinib with recommended doses versus non-upadacitinib therapies.

(A) Serious adverse events; (B) Death; (C) Non-melanoma Skin Cancer; (D) Malignancy excluding non-melanoma skin cancer; (E) Major Adverse Cardiovascular Events; (F) Venous Thromboembolism Events.

Experimental: upadacitinib; Control: Non-upadacitinib including MTX, ADA, etc.

The appearance of the same study is due to subgroup analyses or pooled analyses of different RCTs performed on the same experimental dose and control group with no duplication analyses.

Abbreviations: ADA, Adalimumab; MTX, Methotrexate.

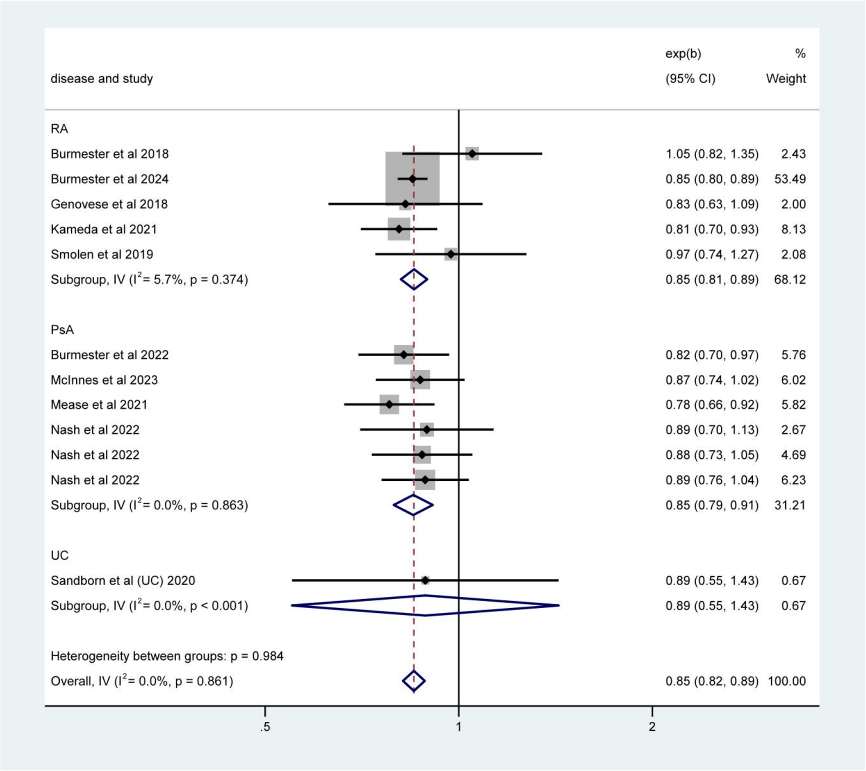

Fig S5. Overall AEs assessed by different doses of upadacitinib.

Experimental: upadacitinib 15 mg QD; Control: padacitinib 30 mg QD.

The appearance of the same study is due to subgroup analyses or pooled analyses of different RCTs performed on the same experimental dose and control group with no duplication analyses.

Abbreviations: QD, once daily.
